# Embryogenic Calli Induction and Salt Stress Response Revealed by RNA-Seq in Diploid Wild Species *Gossypium sturtianum* and *Gossypium raimondii*

**DOI:** 10.3389/fpls.2021.715041

**Published:** 2021-08-25

**Authors:** Hushuai Nie, Yali Wang, Chengcheng Wei, Corrinne E. Grover, Ying Su, Jonathan F. Wendel, Jinping Hua

**Affiliations:** ^1^Laboratory of Cotton Genetics, Genomics and Breeding/Joint Laboratory for International Cooperation in Crop Molecular Breeding, Ministry of Education/Beijing Key Laboratory of Crop Genetic Improvement, College of Agronomy and Biotechnology, China Agricultural University, Beijing, China; ^2^Department of Ecology, Evolution and Organismal Biology, Iowa State University, Ames, IA, United States

**Keywords:** embryogenic callus, salt stress, transcriptome analysis, abscisic acid, transcription factor, *Gossypium raimondii*, *Gossypium sturtianum*

## Abstract

Wild cotton species can contribute to a valuable gene pool for genetic improvement, such as genes related to salt tolerance. However, reproductive isolation of different species poses an obstacle to produce hybrids through conventional breeding. Protoplast fusion technology for somatic cell hybridization provides an opportunity for genetic manipulation and targeting of agronomic traits. Transcriptome sequencing analysis of callus under salt stress is conducive to study salt tolerance genes. In this study, calli were induced to provide materials for extracting protoplasts and also for screening salt tolerance genes. Calli were successfully induced from leaves of *Gossypium sturtianum* (C_1_ genome) and hypocotyls of *G. raimondii* (D_5_ genome), and embryogenic calli of *G. sturtianum* and *G. raimondii* were induced on a differentiation medium with different concentrations of 2, 4-D, KT, and IBA, respectively. In addition, embryogenic calli were also induced successfully from *G. raimondii* through suspension cultivation. Transcriptome sequencing analysis was performed on the calli of *G. raimondii* and *G. sturtianum*, which were treated with 200 mM NaCl at 0, 6, 12, 24, and 48 h, and a total of 12,524 genes were detected with different expression patterns under salt stress. Functional analysis showed that 3,482 genes, which were differentially expressed in calli of *G. raimondii* and *G. sturtianum*, were associated with biological processes of nucleic acid binding, plant hormone (such as ABA) biosynthesis, and signal transduction. We demonstrated that DEGs or TFs which related to ABA metabolism were involved in the response to salt stress, including xanthoxin dehydrogenase genes (*ABA2*), sucrose non-fermenting 1-related protein kinases (*SnRK2*), *NAM, ATAT1*/*2*, and *CUC2* transcription factors (*NAC*), and WRKY class of zinc-finger proteins (*WRKY*). This research has successfully induced calli from two diploid cotton species and revealed new genes responding to salt stress in callus tissue, which will lay the foundation for protoplast fusion for further understanding of salt stress responses in cotton.

## Background

Cotton (genus *Gossypium*) is an agronomically important crop plant consisting of four independently domesticated species (i.e., *G. hirsutum, G. barbadense, G. herbaceum*, and *G. arboreum*) that collectively dominate the natural fiber market. In addition to these important crop species, the genus include over 50 additional species, most of which produce some degree of fiber (Wang et al., 2018). While these species may represent a collective source of fiber improvement genes, wild species also comprise a natural source of resistance to biotic and abiotic stresses, male sterility, and improvement of fiber strength (Fryxell et al., 1992; Grover et al., 2012, 2015; Li et al., 2014; Huang et al., 2017; Wang et al., 2018). Introgression between wild and domesticated cotton is not always straightforward, however, as mating barriers exist among numerous interspecific crosses (Rebeca et al., 2018).

Somatic embryogenesis (SE) and protoplast hybridization are biotechniques used in agriculture to facilitate the exchange of elite and/or desired alleles among species (Li et al., 1991; Przetakiewicz et al., 2003; Navrátilová, 2004; Sakhanokho and Rajasekaran, 2016), including cotton (Wang et al., 2008). In general, protoplasts can be isolated from different explants, such as leaves, hypocotyls, young roots, embryogenic callus, etc. Among them, callus culture is not restricted by geographical and environmental conditions in wild cotton species, and callus can be proliferated continuous and obtained numerous materials for protoplast extraction. Somatic embryogenesis is a process by which plants are regenerated from non-germline tissue, either directly (clonal) or indirectly through the formation of a callus. Plant calluses (or calli) are growing masses of cells that may be derived from various explants, including leaves, hypocotyls, young roots, etc. Embryogenic callus induction (subsequent to the protoplast formation/fusion) is a complex multifactorial system that can be affected by both the endogenous hormones and the exogenous plant growth regulators (PGRs) used to facilitate the callus formation (Nascimento-Gavioli et al., 2017). Therefore, our ability to induce and to successfully develop embryonic calluses for a diversity of species comprises key steps in generating interspecific hybrids through protoplast fusion/plant regeneration (Sun et al., 2005).

Cotton species are known to be difficult for regenerating plantlets, due to the length of the procedure and the variety of limitations encountered (Xu et al., 2013). Although SE and plant regeneration in cultivated *G. hirsutum* and *G. barbadense* have been successful (Xu et al., 2013; Yang et al., 2014; Zheng et al., 2014; Liu et al., 2017; Pandeya et al., 2017; Wei et al., 2019), the conditions necessary to culture and regenerate wild cotton have been explored for few species, i.e., *G. davidsonii, G. klotzschianum, G. stocksii, G. nelsonii, G. gossypioides*, and *G. tomentosum* (Tan and Qian, 1988; Sun et al., 2003, 2006; Yan et al., 2010; Zhang et al., 2010; Sun and Hua, 2020).

Previous reports have shown that the success of cotton embryogenesis callus induction may be determined by genotype (Yang and Zhang, 2010), status of explants (Trolinder and Goodin, 1987; Trolinder and Xhixian, 1989), and media constituents (Trolinder and Goodin, 1988), including plant growth regulators (PGRs) (Sun et al., 2003; Sakhanokho et al., 2004; Ree and Guerra, 2015), nitrogen sources (Cangahuala-Inocente et al., 2014), and inorganic ions (Kumar et al., 2015; Cheng et al., 2016). In general, glucose was the best carbon source used to induce embryogenesis and callus formation, vastly outperforming sucrose (Babbar and Gupta, 1986). Although 2,4-dichlorophenoxyacetic acid (2,4-D, a synthetic auxin) and kinetin (KT) could induce tissue cultures, the application of little to no 2,4-D was better for embryogenic culture production (Sun et al., 2003).

Salt stress is one of the most serious abiotic stresses, capable of causing ion toxicity, oxidative damage, and water deficits through the destruction of ion and osmotic homeostasis, which further affect photosynthesis, metabolic processes, and cellular structures in plants (Gill and Tuteja, 2010; Juknys et al., 2012). Although cotton is known for salt tolerance, increasing salt stress due to climate change and agricultural practices inhibits cotton growth and decreases the yield. The completion of genome sequencing in *G. raimondii* (Paterson et al., 2012; Wang et al., 2012, 2020; Udall et al., 2019), *G. arboreum* (Li et al., 2014; Huang et al., 2020), *G. hirsutum* (Li et al., 2015; Zhang et al., 2015; Hu et al., 2019; Wang et al., 2019; Huang et al., 2020), and *G. barbadense* (Liu et al., 2015; Yuan et al., 2015; Hu et al., 2019; Wang et al., 2019) offers new resources to explore salt stress responses in cotton. Recent research has revealed that transcription factors, such as *WRKY, NAC, ERF*, and *DREB*, are critical to salt tolerance. These transcription factors are thought to regulate the expression patterns of downstream genes and further influence the level of salt tolerance in plants. In addition, dynamic changes under salt stress of hormone signaling, such as abscisic acid (ABA), jasmonate (JA), gibberellic acid (GA), ethylene, and brassinosteroid (BR), also regulate the transcriptional level of stress response genes in plants (Deinlein et al., 2014). Overexpression or knockdown of these genes, such as CBL-interacting protein kinase gene (*CIPK6*) and *SnRK2.6a*, could improve salt tolerance in transgenic cotton (He et al., 2013; Su et al., 2017b, 2020; Guo et al., 2019).

Wild cotton species contain many excellent agronomic traits, including their response to salt stress (Wei et al., 2017; Guo et al., 2019). Phenotypic analyses of salt-stressed plants, for example, have identified wild diploid cotton species with better salt tolerance than cultivated accessions (Dong et al., 2020), which makes introgression of that trait into cultivated cotton desirable; however, natural barriers to hybridization reduce our ability to introgress traits such as salt tolerance. Methods such as protoplast fusion and somatic embryogenesis are suitable options for breeding programs involving wild cotton species, although these are reliant on the establishing methods for somatic embryogenesis for each cotton species.

Here, we describe the first set of successful methods for inducing embryogenic calli in *G. sturtianum* and *G. raimondii*. Calli were obtained from the leaves of *G. sturtianum* and hypocotyls of *G. raimondii* on MSB_5_ medium (Murashige and Skoog + B5 vitamins) supplied with 0.10 mg·L^−1^ 2, 4-D (synthetic auxin), 0.10 mg·L^−1^ KT (kinetin), and 0.10 mg·L^−1^ IAA (auxin) or 0.60 mg·L^−1^ 2, 4-D and 0.25 mg·L^−1^ KT, respectively. In addition, we performed transcriptomic analysis of calli over 48 h (0, 6, 12, 24, and 48) in response to 200 mM NaCl stress to evaluate whether salt stress responses in calli were similar to the patterns reported in whole plants, thereby bypassing the need to grow the entire plant prior to abiotic stress screening. In general, we found that, as previously reported for cotton, salt stress resulted in differential gene expression of many transcription factors and ABA-related genes for both *G. sturtianum* and *G. raimondii*. Our findings lay the foundation for establishing an efficient protocol to prepare materials for protoplast fusion and somatic hybridization and provide valuable information for cotton salt tolerance and abiotic stress research using calli that may be useful in germplasm improvement and cotton breeding.

## Materials and Methods

### Plant Materials, Callus Induction, and Proliferation

The plant materials in this study were two diploid wild cotton species, *G. sturtianum* (C_1_) and *G. raimondii* (D_5_, Paterson et al., 2012; Wang et al., 2012; Udall et al., 2019). Seeds were delinted and surface-sterilized with 70% (w/v) alcohol for 30 sec, then immersed in 0.1% (w/v) HgCl_2_ for 10 min followed by five rinses with sterile ddH_2_O. The sterilized cotton seeds were germinated and grown on 1/2 MS (Murashige and Skoog, pH 5.9) with 7.5 g·L^−1^ agar and 20 g·L^−1^ sucrose in the dark for 7 days at 28 ± 2°C. Hypocotyls and leaves of the seedlings were cut into sections (0.5–1.0 cm each) or small pieces (1 cm^2^) as explants for callus induction.

The callus induction medium for both species was MSB_5_, i.e., MS medium plus B_5_ vitamins (Lommen et al., 2017), supplemented with additional plant hormones and growth regulators. For *G. sturtianum*, the MSB_5_ media were supplemented with 30 g/L glucose, 2.7 g·L^−1^ phytagel, 0.1 mg·L^−1^ 2,4-D, 0.1 mg·L^−1^ KT, and 0.10 mg·L^−1^ IAA. For *G. raimondii*, the hormone concentrations suitable for initiating the callus formation were 0.60 mg·L^−1^ 2,4-D and 0.25 mg·L^−1^ KT. In both species, calli were cultured at 28 ± 2°C with a 16-h photoperiod. After 4 weeks of growth in the induction media, fresh, loose, and yellow-green non-embryogenic calli were transferred into MSB_5_ medium supplemented with different ratios of 2,4-D and KT.

### Embryogenic Callus Induction and Maintenance

The calli of *G. sturtianum* and *G. raimondii* were transferred to media containing 3% (w/v) sugar (glucose or sucrose) for three to four subcultures (one per month). Different PGRs and nitrogen sources were added to the medium to induce embryogenic callus formation in *G. sturtianum* (Table 1, Supplementary Table 1) and *G. raimondii* (Supplementary Table 2). Potential embryogenic calli of *G. sturtianum* and *G. raimondii* were selected and transferred into liquid media, which was identical to the solid MSB_5_ media for each species, but without the phytagel and with the addition of 1.00 g·L^−1^ glutamine and 0.50 g·L^−1^ asparagine (Supplementary Table 3). For each species, about 1–2 g of friable non-embryogenic calli was selected for liquid culture (flasks with 40 ml liquid media), which were shaken at 120 rpm under a 16/8-h light/dark cycle at 28°C. The larger cell masses were removed every 1–2 days, and the culture media were replaced every 7 days.

**Table 1:** Effects of PGR combinations on embryonic callus induction of *G. sturtianum*.

**Medium**	**Hormone combination (mg·L^**−1**^)**	**Nitrogen source (g·L^**−1**^)**	**Callus description**
C1	(0.20) 2,4-D + (0.10) KT	(3.80) KNO_3_	Yellow, fast proliferation, embryogenic
C2	(0.20) 2,4-D + (0.10) KT	(1.90) KNO_3_	Yellow, fast proliferation, embryogenic
EI1	(0.20) 2,4-D + (0.10) KT + (0.20) IBA	(1.65) NH_4_NO_3_ + (1.90) KNO_3_	Yellow, slow proliferation
EI2	(0.20) 2,4-D + (0.10) KT + (0.30) IBA	(1.65) NH_4_NO_3_ + (1.90) KNO_3_	Yellow, fast proliferation, embryogenic
EI3	(0.20) 2,4-D + (0.10) KT + (0.40) IBA	(1.65) NH_4_NO_3_ + (1.90) KNO_3_	Gray, slow proliferation
EI4	(0.20) 2,4-D + (0.20) KT + (0.40) IBA	(1.65) NH_4_NO_3_ + (1.90) KNO_3_	Gray, fast proliferation, non-embryogenic
EI5	(0.20) 2,4-D + (0.20) KT + (0.50) IBA	(1.65) NH_4_NO_3_ + (1.90) KNO_3_	Yellow, fast proliferation, embryogenic
EI6	(0.20) 2,4-D + (0.20) KT + (0.60) IBA	(1.65) NH_4_NO_3_ + (1.90) KNO_3_	Yellow, fast proliferation, non-embryogenic and embryogenic coexistence
EI7	(0.20) 2,4-D + (0.10) KT + (0.30)IBA	(3.80) KNO_3_	Yellow, fast proliferation, embryogenic

### Salt Stress Treatments, Sampling, and RNA Sequencing

The resulting embryogenic calluses of *G. raimondii* and *G. sturtianum* were treated with 200 mM NaCl after they were grown in culture for 21 days. Three biological replicates were harvested for each sample at various timepoints (0, 6, 12, 24, and 48 h) after exposure to salt stress. Total RNA of each sample was isolated using a modified CTAB method according to the instruction manual (Zhao et al., 2012), and 1 μg RNA per sample was used as input material for cDNA synthesis. The first-strand cDNA synthesis was accomplished using random hexamer primers and M-MuLV Reverse Transcriptase (RNase H^−^); then the second-strand synthesis was accomplished using DNA polymerase I and RNase H to generate the final cDNAs. These cDNAs were subjected to end-repair/dA-tail (DNA A-Tailing Kit, TaKaRa) and adaptor ligation, and the resulting libraries were sequenced on the Illumina Novaseq platform as 150-bp paired-end reads with 6 × depth.

### Quality Control, Mapping, and Quantification of Gene Expression Levels

A total of 1,631 million raw reads (accession number: PRJNA736855) were first processed through in-house Perl scripts (Supplementary Table 4), which removed adapter sequences, poly-N reads, and low-quality reads from raw data. The resulting clean reads were mapped to the cotton genome (*G. raimondii*, Paterson et al., 2012) using Tophat2.2.0 (Kim et al., 2013), resulting in ~97% and 78–83% of the clean reads per sample successfully mapping to the *G. raimondii* reference genome from the *G. raimondii* and *G. sturtianum* samples, respectively, 94% and 76–80% of which were uniquely mapping (Supplementary Table 5). FPKM (fragments per kilobase of exon per million fragments mapped) was calculated for each gene using Cufflinks (Kim et al., 2013). Correlation coefficient and principal component analysis (PCA) of the gene expression showed that three biological replicates of each sample have good reproducibility and high reliability, indicating that these sequencing data could be used for differential gene expression (Supplementary Figures 1A,B).

### Gene Differential Expression and Functional Analysis

Differential expression analysis of two groups (three biological replicates per condition) was performed using the DESeq2 R package (1.20.0). Only genes with an absolute value of log2 ratio ≥ 1 and FDR significance score <0.01 were considered differentially expression genes (DEGs). Gene ontology (GO) annotation of DEGs was obtained *via* the Blast2GO program (Götz et al., 2008), and subsequent visualization, comparisons, and plotting of GO annotations were performed using OmicShare tools, a free online platform for data analysis (http://www.omicshare.com/tools). The output of enrichment was limited to FDR < 0.05. KEGG pathway analysis was performed through alignment against KEGG (https://www.genome.jp/kegg/).

### Quantitative Real-Time PCR

Quantitative real-time PCR was performed using SYBR Green PCR Master mix according to the manufacturer's instructions. The constitutively expressed gene *GhUBQ7* was used for the normalization of gene expression (Nie et al., 2018). All reactions were run three times, and the average number of threshold cycle (Ct) values was produced automatically by the qRT-PCR amplifier. The relative gene expression of each transcript was normalized against the reference gene *GhUBQ7* using the 2^−ΔΔCt^ method (Livak and Schmittgen, 2001).

## Results

### Embryogenic Callus Induction and Proliferation

**Figure 1:**
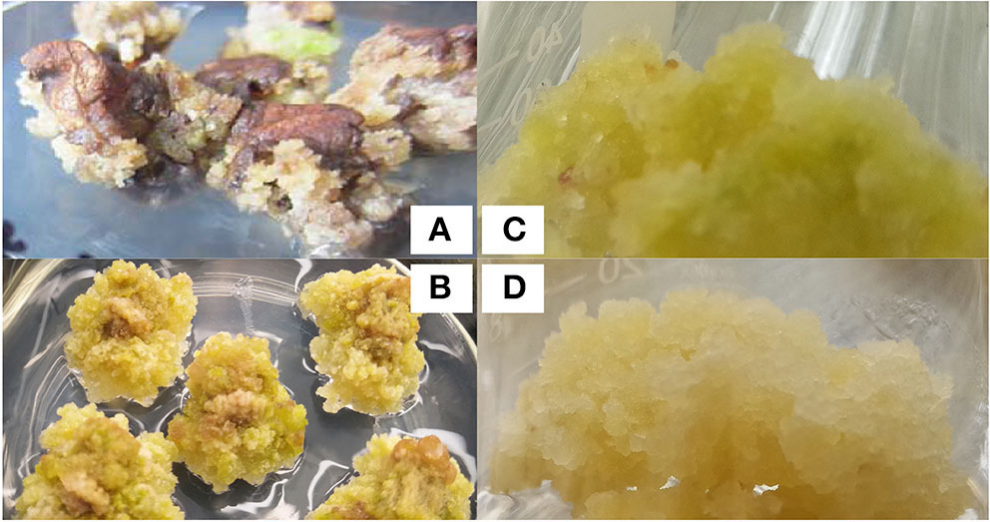
Embryogenic callus induction in two diploid wild cotton species. **(A)** Callus induced from *G. sturtianum*, **(B)** callus induced from *G. raimondii*. **(C)** embryogenic callus induced from *G. sturtianum*, **(D)** embryogenic callus induced from *G. raimondii*.

Calli of leaves in *G. sturtianum* were induced on MS basal medium supplemented with 0.10 mg·L^−1^ 2, 4-D, 0.10 mg·L^−1^ KT, and 0.10 mg·L^−1^ IAA, whereas *G. raimondii* had slightly different induction conditions (i.e., MS basal medium with 0.60 mg·L^−1^ 2, 4-D, 0.25 mg·L^−1^ KT). Notably, the colors and textures of the calli from each species were different. Hypocotyls were used for *G. raimondii*, which produced a greater number and more friable calli than the cotyledons used for *G. sturtianum*. The phenotypic characteristics of these calli are shown in Figures 1A,B. While the original calli for both *G. sturtianum* and *G. raimondii* were loose, slight color differences were observable (i.e., yellow-green vs. light yellow, for *G. sturtianum* and *G. raimondii*, respectively).

Embryogenic calli induction also required different conditions for each species. Callus proliferation progressed rapidly during continuous subculture, becoming friable after 3–4 months and with a tendency to differentiate embryogenic callus. Calli from *G. sturtianum* were light yellow and loose under N or C starvation conditions, and these were selected for subculture on media lacking either carbon or nitrogen sources to induce embryogenic callus formation. Although *G. sturtianum* calli were gray under nitrogen deficiency, they grew rapidly, particularly in N1 and N3 media (Supplementary Table 1); however, when grown in media lacking a carbon source, calli were green and slow to proliferate, indicating that embryogenic callus induction was more successful under nitrogen deficiency. These calli were subsequently transferred to either N4, N5, N6, or N7 medium (Supplementary Table 1), all of which typically produced light-yellow embryogenic calli (Figure 1C). In contrast, *G. raimondii* embryogenic calli were most successfully induced under a double concentration of KNO_3_, with a 2–3 ratio of IBA/KT as most appropriate (Figure 1D). As expected, sucrose was better than glucose at promoting the formation of embryogenic calli for *G. raimondii* (see Supplementary Table 2 for the effects of each media combination on the induction of embryogenic callus).

Successful retrieval of EC was different for each species. Throughout a month-long suspension culture, the calli and liquid media of *G. raimondii* remained relatively bright, with smaller cell masses having uniform size and synchronous growth (Supplementary Figure 2A). Subsequent transfer to a solid form of the same media allowed the suspension culture cell masses to gradually develop into brightly colored embryogenic calli with a loose texture (Supplementary Figure 2B). Conversely, the liquid media for *G. sturtianum* calli induction tended to oxidize, resulting in quick browning, with most calli dying after transfer to solid medium (Supplementary Figures 2C,D), suggesting further modifications to the *G. sturtianum* EC induction media were necessary.

### Critical Factors Affecting Proliferation and Differentiation of *G. sturtianum* Embryogenic Calli

**Figure 2:**
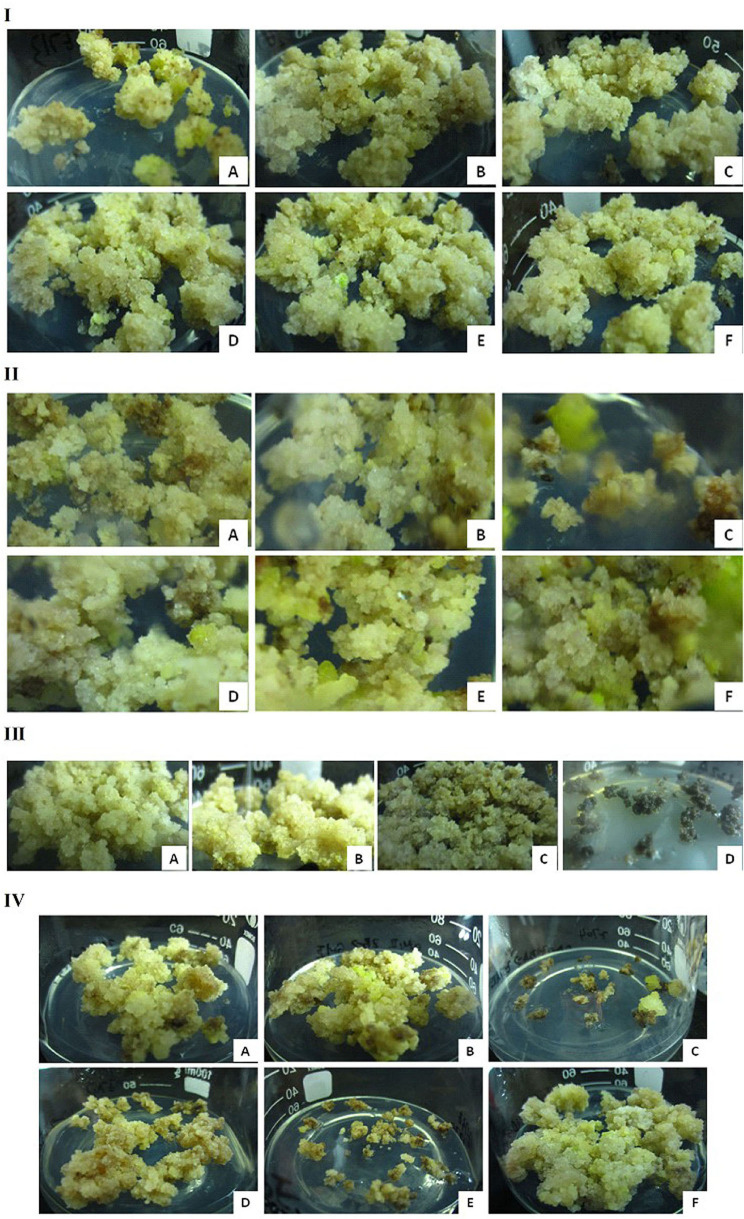
Embryogenic callus initiation and proliferation of *G. sturtianum*. **(I)** IBA and KT, **(A–F)** EI1-EI6 corresponding to Table 1; **(II)** nitrogen source, **(A–C)** C1, C2, C3 corresponding to Supplementary Table 6 **(D–F)** EI7, E1, EI9 corresponding to Table 1 and Supplementary Table 6; **(III)** FeSO_4_, **(A–D)** C1, E4, E6, E7 corresponding to Supplementary Table 7; **(IV)** CuSO_4_, **(A,B)** C1 corresponding to Supplementary Table 7; **(C–F)** E7–E10 corresponding to Supplementary Table 7.

Because different plant growth regulator (PGR) combinations are important for the proliferation and differentiation of embryogenic calli (Sakhanokho et al., 2004; Ge et al., 2017), optimization is often required for EC induction and proliferation. In the initial EC induction, we found that *G. sturtianum* did not require a nitrogen source (here, NH_4_NO_3_; Table 1), but that the addition of IBA was conducive to the proliferation of the embryonic callus at an optimal IBA/KT ratio of 3 (on MSB_5_ media; Figure 2I). These results suggest that uncoordinated proportions of hormones can easily lead to the reversal of EC into non-embryonic calli.

To further refine our understanding of the nutrient and hormone requirements for *G. sturtianum* embryonic calli induction, we evaluated EC induction on the basic media containing different concentration gradients of KNO_3_ and supplemented with different PGRs combinations (Supplementary Table 6, Figure 2II). High concentrations of KNO_3_ combined with low concentrations of PGRs resulted in embryogenic calli that subsequently turned brown and died (Figure 2II). When the PGR concentrations were doubled, the inhibition associated with high concentrations of KNO_3_ was eliminated; however, the resulting calli exhibited no embryogenicity. Only those calli that were grown in media containing 7.60 g·L^−1^ KNO_3_ with lower concentrations of KT and with the addition of IBA resulted in embryogenic calli, suggesting that IBA may facilitate EC proliferation.

The concentrations of FeSO_4_ and CuSO_4_ also greatly affected the growth rate and yield of embryonic calli in *G. sturtianum*. Most of the calli in media containing 1-2x concentrations of FeSO_4_ (E4) resulted in EC that were light-yellow and soft with fast proliferation (Figure 2III), suggesting that these FeSO_4_ concentrations (optimally 1.5–2.0x) promote embryonic callus proliferation and differentiation (Supplementary Table 7). Notably, low concentrations of FeSO_4_ resulted in callus death. Conversely, both high and zero concentrations of CuSO_4_ performed non-optimally. Calli grown on media with high CuSO_4_ (both with and without 2,4-D; Supplementary Table 7) either rapidly died or were brown and slow to proliferate, remaining non-embryogenic and ultimately becoming dying (Figure 2IV). Calli grown in the absence of CuSO_4_ were equally unusable, either rapidly dying (if 2,4-D was excluded from the media) or proliferating well but remaining non-embryogenic (in the presence of 2,4 D; Supplementary Table 7). Together, these results suggest that a slight elevation of FeSO_4_ and a standard concentration of CuSO_4_ are suitable for EC generation.

### Characterization of EC DEGs in Response to Salt Stress

**Figure 3:**
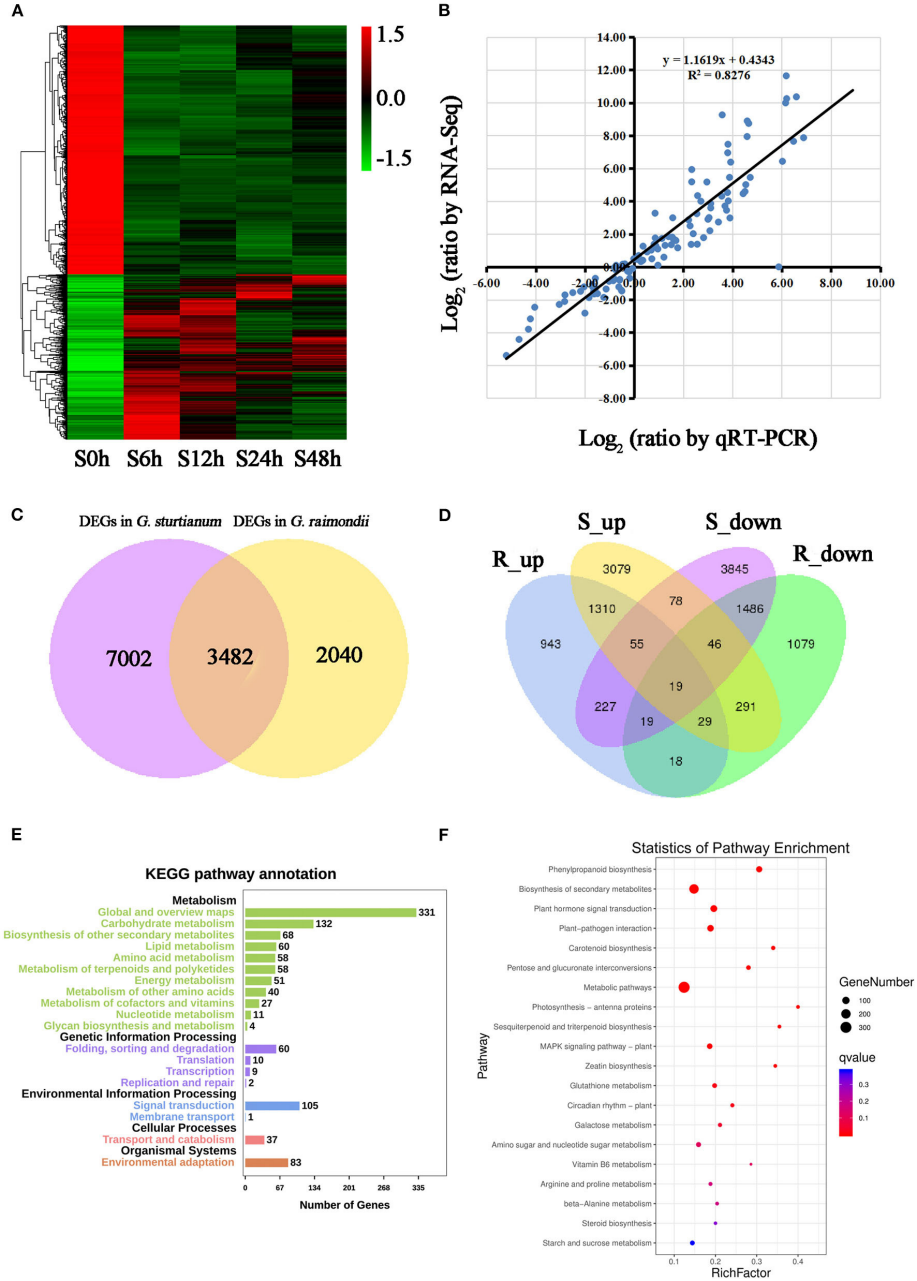
Identification and functional analysis of differently expressed genes during different stages under salt stress in *G. raimondii* and *G. sturtianum*; **(A)** Heatmap of differential expression during diverse phases under salt stress in *G. sturtianum*; **(B)** Correlation analysis of gene expression profiles between RNA-Seq and qRT-PCR; **(C)** Venn diagrams of all DEGs of *G. raimondii* and *G. sturtianum*; **(D)** Venn diagrams of upregulated and downregulated DEGs of *G. raimondii* and *G. sturtianum*; **(E,F)** KEGG enrichment analysis of common DEGs in *G. raimondii* and *G. sturtianum*. S0h, Callus of *G. sturtianum* under salt stress for 0 h; S6h, Callus of *G. sturtianum* under salt stress for 6 h; S12h, Callus of *G. sturtianum* under salt stress for 12 h; S24h, Callus of *G. sturtianum* under salt stress for 24 h; S48h, Callus of *G. sturtianum* under salt stress for 48 h (both here and below). R_up, Upregulated genes under salt stress in *G. raimondii*; R_down, Downregulated genes under salt stress in *G. raimondii*; S_up, Upregulated genes under salt stress in *G. sturtianum*; S_down, Downregulated genes under salt stress in *G. sturtianum*.

Generation of EC is often promoted to protoplast fusion to generate hybrids between species/accessions that have natural reproductive barriers, which is particularly useful in agricultural breeding programs. Accordingly, it may be useful to understand whether responses to stress can be characterized in the calli themselves. Here, we explore the gene expression patterns of calli in *G. sturtianum* and *G. raimondii* under salt stress to determine if the abiotic stress response in calli is similar to that previously described for cotton plants. Toward this end, transcriptome sequencing was performed on calli treated with 200 mM NaCl and sampled at 0, 6, 12, 24, and 48 h, respectively; the direction of regulation (i.e., upregulated or downregulated) here refers to the comparison with 0-h salt treatment. Differential gene expression was detected in 5,522 genes in *G. raimondii* and 10,484 genes in *G. sturtianum*, with the majority of DEG exhibiting expression changes during the first two timepoints. The number of DEG peaked at 12 h post-exposure (3,552 in *G. raimondii* and 6,917 in *G. sturtianum*), subsequently returning to the lower levels. These observations suggest that the initial timepoints after salt stress are critical for the calli to respond to stress. This changing trend is similar to the DEGs in roots and leaves in cotton under salt stress (Su et al., 2020). It is also worth noting that the number of downregulated DEGs was greater than that of the upregulated DEGs in each salt stress period for both species, indicating that most genes are negatively regulated in response to salt stress (Figure 3A; Supplementary Figure 1C).

Among the 5,522 DEGs of *G. raimondii*, 850 were differentially expressed during all four periods after salt stress (Supplementary Figure 3A), and there were 508, 531, 418, and 420 genes that were specifically differently expressed at the stage of salt stress 6, 12, 24, and 48 h, respectively. Similarly, among the 10,484 DEGs of *G. sturtianum*, 1,207, 694, 646, and 716 genes were specifically differentially expressed at the 6, 12, 24, and 48 h after salt stress, respectively, with 2,583 common DEGs in each stage (Figure 3A; Supplementary Figure 1D). In order to validate the reliability of RNA sequencing, qRT-PCR was carried out to detect the expression patterns of DEGs in different samples. Fourteen genes were randomly selected (Supplementary Table 8), whose abundance confirms the general agreement between quantitative RT-PCR measurements and estimates of abundance from transcriptome sequencing (R^2^ = 0.8276; Figure 3B; Supplementary Figure 3B).

In total, 12,524 DEGs were collectively detected in *G. sturtianum* and *G. raimondii* during salt stress. From this pool of DEGs, 3,482 were identified as differentially expressed in both species (Figure 3C), with 1,310 upregulated in both and 1,486 downregulated in both (Figure 3D). Functional annotation of these shared DEGs (based on GO ontology) identified GO categories for 2,552 genes that mainly included the categories Biological Process, Molecular Function, and Cellular Component and included 54 secondary GO functional classifications (Supplementary Figure 3C). We also employed the KEGG pathway analysis to predict important metabolic processes and networks operating during salt stress in cotton calli. Fewer shared DEGs (1,147) were annotated by KEGG, which were mainly divided into five categories: metabolism, genetic information processing, environmental information processing, cellular processes, and organismal systems (Figure 3E). Most (73.23%) of the genes were involved in metabolic pathways, and they were mainly enriched in the subcategories metabolic pathways (310), biosynthesis of secondary metabolites (201), plant–pathogen interaction (69), and glutathione metabolism (65). In addition, the biosynthetic pathways of some hormones, such as carotenoid biosynthesis pathways related to abscisic acid synthesis (16), cysteine and methionine metabolic pathways related to ethylene synthesis (cysteine and methionine metabolism, 20), zeatin biosynthesis related to cytokinin synthesis (zeatin biosynthesis, 10), and one pathway for “plant hormone signal transduction” (78) (Figure 3F) were also prominent salt stress response DEGs. The combined results of GO and KEGG enrichment analysis suggest that genes related to hormone signal transduction and nucleic acid binding were significantly affected under salt stress in cotton callus. These results were consistent with previous research, which identified salt tolerance genes in cotton roots and leaves (Rodriguez-Uribe et al., 2011; Shi et al., 2015).

### Abscisic Acid Metabolic Pathway Is Very Important to Respond to Salt Stress in Cotton Callus

**Figure 4:**
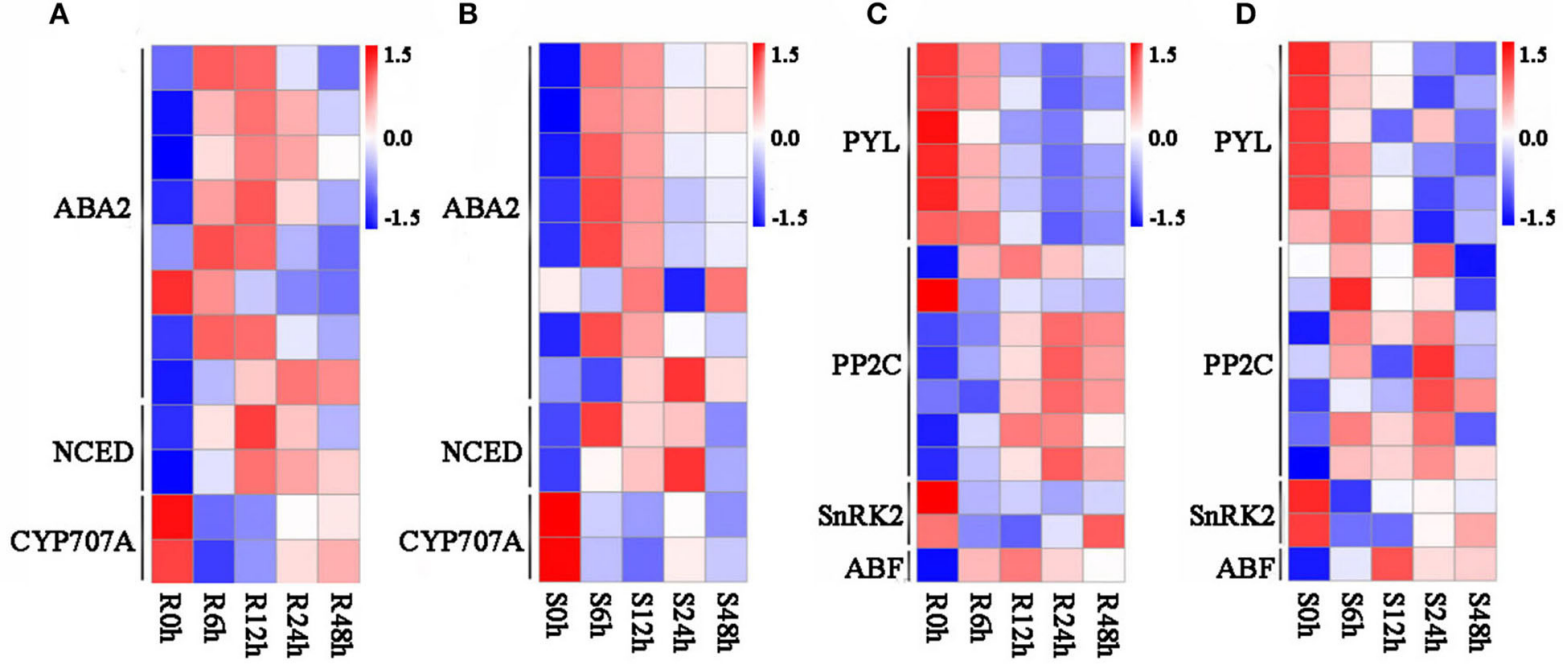
Expression pattern of genes related to ABA biosynthesis and signal transduction. **(A)** Expression pattern of genes related to ABA biosynthesis in *G. raimondii*; **(B)** expression pattern of genes related to ABA biosynthesis in *G. sturtianum*; **(C)** expression pattern of genes related to ABA signal transduction in *G. raimondii*; **(D)** expression pattern of genes related to ABA signal transduction in *G. sturtianum*. R0h, Callus of *G. raimondii* under salt stress for 0 h; R6h, Callus of *G. raimondii* under salt stress for 6 h; R12h, Callus of *G. raimondii* under salt stress for 12 h; R24h, Callus of *G. raimondii* under salt stress for 24 h; R48h, Callus of *G. raimondii* under salt stress for 48 h (both here and below).

Abscisic acid is a critical regulatory factor in response to abiotic stress in plants; large numbers of studies have proved that ABA metabolism participates in response to salt stress in plants, such as in roots and leaves (Gong et al., 2020; Su et al., 2020). In our study, lots of common DEGs shared by *G. sturtianum* and *G. raimondii* is significantly enriched in the ABA synthesis and signal transduction pathway. There were 12 DEGs enriched in the carotenoid synthesis pathway, which related to abscisic acid synthesis (Table 2), including eight xanthoxin dehydrogenase genes (*ABA2*), two 9-cis-epoxycarotenoid dioxygenase genes (*NCED*), and two abscisic acid 8′-hydroxylase genes (*CYP707A*). Most of the *ABA2* genes showed similar expression patterns in the two cotton species, with a trend of first up and then down after salt stress; these genes include *Gorai.002G236800, Gorai.002G237000, Gorai.002G237200, Gorai.002G237300, Gorai.002G237400, Gorai.006G151500*, and *Gorai.006G151900* (Figures 4A,B). Two *NCED* genes (*Gorai.002G038100* and *Gorai.004G270800*) were upregulated under salt stress and the abundance grew to the peak at 6 h and 12 h after salt stress, then gradually decreased (Figures 4A,B). Interesting, the expression abundance of two *CYP707A* genes (*Gorai.004G177200* and *Gorai.008G218900*), which are mainly involved in the ABA decomposition process, was downregulated after salt stress (Figures 4A,B).

**Table 2:** List of genes related to ABA biosynthesis and signal transduction.

**Pathway**	**Pathway ID**	**KID**	**Gene family**	**Gene ID**
Carotenoid biosynthesis (ABA)	ko00906	K09841	*ABA2*	*Gorai.002G236800*
		K09841		*Gorai.002G237000*
		K09841		*Gorai.002G237200*
		K09841		*Gorai.002G237300*
		K09841		*Gorai.002G237400*
		K09841		*Gorai.002G237500*
		K09841		*Gorai.006G151500*
		K09841		*Gorai.006G151900*
		K09840	*NCED*	*Gorai.002G038100*
		K09840		*Gorai.004G270800*
		K09843	*CYP707A*	*Gorai.004G177200*
		K09843		*Gorai.008G218900*
Plant hormone signal transduction (ABA)	ko04075	K14496	*PYL*	*Gorai.002G266100*
		K14496		*Gorai.006G185000*
		K14496		*Gorai.007G031800*
		K14496		*Gorai.009G323000*
		K14496		*Gorai.010G194800*
		K14496		*Gorai.011G290300*
		K14497	*PP2C*	*Gorai.001G013500*
		K14497		*Gorai.003G128900*
		K14497		*Gorai.006G201000*
		K14497		*Gorai.008G269800*
		K14497		*Gorai.008G282600*
		K14497		*Gorai.009G096200*
		K14497		*Gorai.012G003400*
		K14498	*SnRK2*	*Gorai.002G006900*
		K14498		*Gorai.011G121900*
		K14432	*ABF*	*Gorai.009G275700*

Another 16 DEGs, including six abscisic acid receptor *PYR/PYL* family genes (*PYL*), seven protein phosphatase 2C genes (*PP2C*), two *SnRK2* genes, and one ABA-responsive element binding factor (*ABF*), were participated in the ABA signaling pathway (Table 2). Among them, six *PYL* genes (*Gorai.002G266100, Gorai.006G185000, Gorai.007G031800, Gorai.009G323000, Gorai.010G194800*, and *Gorai.011G290300*) showed a continuous downward regulation after salt stress in *G. raimondii* (Figure 4C). Interestingly, except for *Gorai.011G290300* with the upregulated expression at 6 h of salt stress in *G. sturtianum*, other five PYL genes present down-regulated trends after salt stress in *G. sturtianum* same as in *G. raimondii* (Figure 4D). The expression pattern of seven *PP2C* genes showed the same trends at different stages after salt stress in *G. sturtianum*, and all of them are upregulated in both 6- and 24-h periods and downregulated at 12 and 48 h. However, they displayed different expression patterns in two cotton species (Figure 4D). *SnRK2* gene *Gorai.011G121900* was downregulated first and then upregulated gradually after 12 h in both cotton species, while the expression abundance of another *SnRK2* gene *Gorai.002G006900* presented continuous downregulation after salt stress (Figures 4C,D).

The MAPK (mitogen-activated protein kinase) signal transduction pathway is conservative and important to stress response, and transgenic technology has proved that MAPK is critical to salt tolerance in many species (Kiegerl et al., 2000; Shi et al., 2009; Su et al., 2020). Among the 3,482 DEGs in callus, 39 ones are related to the MAPK signal pathway, including two MAPKK protein kinases, seven *WRKY/ERF* transcription factors, three *ERF1* genes, and two *WRKY* genes (Supplementary Table 9). *MKK2* gene *Gorai.001G013600* was upregulated after salt stress, maybe positively correlated with a salt stress response. The MAPK signaling pathway interacts with the ABA signaling pathway, and ABA could activate the MAPK signaling pathway by acting as an upstream signaling molecule (Supplementary Figure 4). In this study, 15 DEGs were involved in the ABA-mediated MAPK signaling pathway, which were consistent with the genes involved in the ABA signaling pathway in the above mentioned. These results indicated that salt-tolerance genes related to MAPK signal pathway also participate in ABA signal transduction in cotton callus, this result has been confirmed in other tissues of the plants (Xing et al., 2007; Lin et al., 2017; Su et al., 2020).

### Numerous TFs Are Responsive to Salt Stress in Cotton Callus

**Figure 5:**
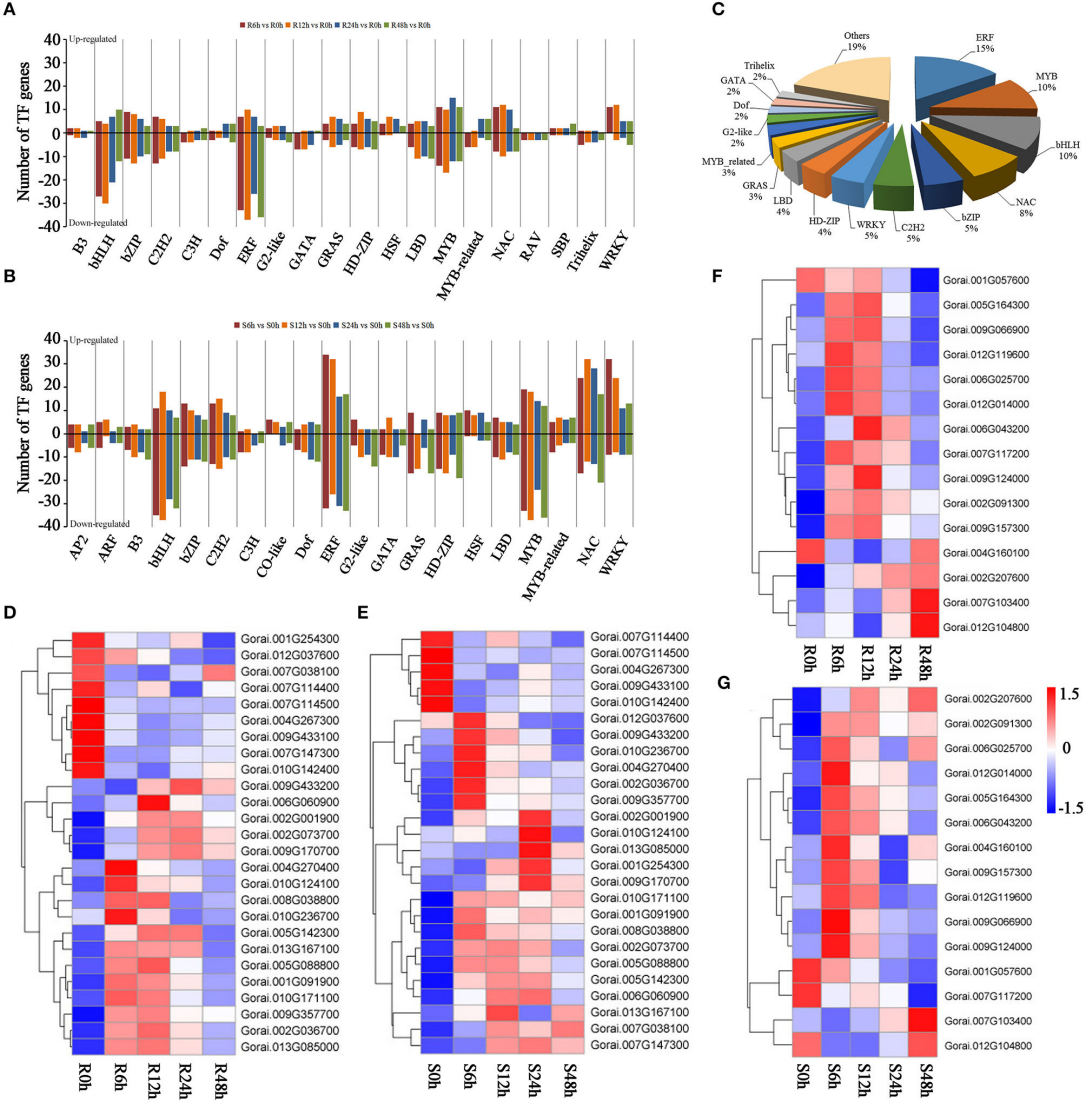
Transcription factors involved in salt tolerance. **(A)** Enrichment analysis of differentially expressed transcription factors in *G. raimondii* under salt stress; **(B)** enrichment analysis of differentially expressed transcription factors in *G. sturtianum* under salt stress; **(C)** statistics of common transcription factors in *G. raimondii* and *G. sturtianum*; **(D)** expression pattern of *NAC* TFs in *G. raimondii*; **(E)** expression pattern of *NAC* TFs in *G. sturtianum*; **(F)** expression pattern of *WRKY* TFs in *G. raimondii*; **(G)** expression pattern of *WRKY* TFs in *G. sturtianum*.

Many of the TF families were shown to be responsive to salt stress, and most of these might enhance salt tolerance through ABA signaling. Members of *ERF, bHLH, MYB, bZIP*, and *C2H2* families are downregulated under salt tolerance in *G. raimondii* (Figure 5A). However, most of the differentially expressed TFs in *G. sturtianum*, such as *ERF, bHLH, MYB, WRKY*, and *NAC*, are upregulated after salt stress (Figure 5B). These results indicate that transcription factor responses to salt stress have species and spatiotemporal specificity.

Of the 3,482 DEGs, 327 TFs were shared by *G. raimondii* and *G. sturtianum*, belonging to 40 TF families. Among these, *ERF, MYB, bHLH, NAC, bZIP, C2H2*, and *WRKY* families account for a large proportion (Figure 5C), suggesting that these transcription factors may play a role in salt stress response. In our study, 26 and 16 DEGs were annotated in the *NAC* and *WRKY* families, respectively, most upregulated after salt stress (Figures 5D–G). Previous research has shown that members of the *NAC* family are involved in ABA signaling (Chen et al., 2014). It is worth noting that there were 17 *NAC* family genes that were upregulated in *G. raimondii* (Figure 5D), and 21 genes were upregulated in *G. sturtianum* (Figure 5E). In addition, some *NAC* genes, such as *Gorai.001G091900, Gorai.002G036700, Gorai.004G270400, Gorai.009G357700, Gorai.010G171100*, and *Gorai.009G170700*, were both significantly upregulated in the two cotton varieties under salt stress. For the *WRKY* family, most genes were upregulated and expression was highest at 6 h or 12 h after salt stress in both *G. raimondii* and *G. sturtianum* (Figures 5F,G). The above results indicate that the members of the *NAC* and *WRKY* family are induced to be differentially expressed by salt stress, and their expression patterns are similar in the two cotton species, suggesting that they play a role in response to salt stress.

## Discussion

### Embryogenic Callus Induction Provides a Material Basis for Protoplast Fusion in Wild Cotton

There are many outstanding and valuable characteristics among wild species in cotton breeding programs; however, reproductive isolation among species is an obstacle to producing hybrid plants through conventional breeding. Therefore, it is essential to develop the resources for somatic cell regeneration of wild cotton species, which will lay the foundation for the creation of new germplasm resources through somatic hybridization of cultivated and wild cotton and subsequent regeneration. Protoplast fusion technology for somatic cell hybridization is an advantageous way to obtain interspecific hybrids, which operates through fused protoplasts derived from embryogenic calli and somatic embryos. A current limitation to this technology in cotton is that the growth and development of wild cotton species require specific environmental conditions, which make it difficult to breed these species and/or obtain a suitable number of materials for protoplast isolation. Therefore, our ability to cultivate and maintain calli from diverse wild cotton species will provide the materials necessary for protoplast separation and subsequent fusion. Presently, only a few reports related to embryogenesis callus and plant regeneration in wild cotton species are available (Sun et al., 2003, 2006; Yan et al., 2010), none of which are broadly applicable to other cotton species. Therefore, suitable induction systems for embryogenic calli must be individually established for different wild cotton species.

In general, differentiating embryogenic calli from non-embryogenic calli is challenging, and insufficient methods exist for most cotton species. The phenotypic characteristics of embryogenic calli from wild cotton species are similar to those found in upland cotton, i.e., they are friable, cream-colored, granular, and yellow-green (Sakhanokho et al., 2004; Zhang Y. et al., 2014). Here we used existing knowledge regarding EC induction in cotton and other plants to circumscribe a set of conditions with potential for EC induction in the wild cotton species *G. sturtianum* and *G. raimondii*. In general, we found that the required combinations of PGRs and nutrients were similar between *G. sturtianum* (0.10 mg·L^−1^ KT + 0.20 mg·L^−1^ 2,4-D) and *G. raimondii* (0.60 mg·L^−1^ 2, 4-D, 0.25 mg·L^−1^ KT), with deviations producing slower-growing, brown, or fatal results. It is clear that the concentration of 2,4-D plays a role in EC generation and that we need to gradually reduce the concentration of 2,4-D during subsequent subcultures. Explants were also found to influence the embryogenic calli production, with hypocotyls producing more friable calli than cotyledons, as previously noted (Chee et al., 1990). In addition, different concentrations of nitrogen sources supplemented in media also influence both viability and conversion from non-embryogenic to embryogenic calli (Loukanina and Thorpe, 2007).

Because alternating solid and liquid cultures can effectively shorten the time for embryogenic callus induction, as demonstrated in upland cotton (Rajasekaran, 1996), generating suitable growth conditions for this alternating procedure is advantageous for improving the pace of research and breeding. As with EC induction in general, however, it is difficult to induce embryogenic calli for diploid wild cotton in a solid-liquid alternate culture. Although we were able to improve the efficiency for embryogenic callus induction in *G. raimondii* based on suspension cultures, solid–liquid alternate cultures, and other methods, this strategy is not suitable for *G. sturtianum* embryogenic callus induction. In general, *G. sturtianum* EC induction was more challenging and further exploration is needed.

### In Callus, DEGs Related to Salt Tolerance Also Played Key Roles in Whole Cotton Plants

At present, most studies about callus responses to salt stress focused on physiological indicator detection (Badawy et al., 2008), with few studies on the transcriptome of cotton callus in response to salt stress. Here, we obtained 5,522 and 10,484 genes that were differentially expressed by salt stress in *G. raimondii* and *G. sturtianum*, respectively. There were two reasons for much more DEGs in *G. sturtianum* than in *G. raimondii*; the first one is that the callus of *G. sturtianum* is more sensitive to salt stress than the callus in *G. raimondii*. Another reason is that the genome sequence of *G. sturtianum* (C1) has not been released; using the genome of *G. raimondii* (D5) as a reference for *G. sturtianum* RNA-Seq analysis will affect the analytical results. A total of 3,482 genes were differentially expressed under salt stress both in *G. sturtianum* and *G. raimondii* callus. These DEGs were enriched in the biological processes of cellular process, biosynthesis of secondary metabolites, plant hormone biosynthesis, signal transduction, and other metabolic pathways. These results imply the rapid response of secondary metabolites and plant hormones metabolism under salt stress, and a similar response mechanism also existed in cotton roots and leaves (Rodriguez-Uribe et al., 2011; Shi et al., 2015; Su et al., 2020). Furthermore, some transcription factor families such as *NAC, WRKY, MYB*, and a large number of genes that are implicated in ABA biosynthesis, ABA signaling transduction, and MAPK signaling pathway were also differentially expressed under salt stress in callus. These biological processes or TF families have been shown to play an important role in the response of whole cotton plants to salt stress (Golldack et al., 2011; Zhang et al., 2016; Wei et al., 2017; Su et al., 2020). *G. aridum, G. davidsonii*, and *G. klotzschianum* have strong salt tolerance, and the functional genes related to reactive oxygen species, plant hormones, and transcription factors were induced under salt stress in these species (Wei et al., 2017). Most of the differentially expressed genes isolated from *G. aridum* under salt stress were homologous to plant stress resistance genes, and 24% of them were related to metabolic processes. Overexpression of *GarMSL* and *GarCYP* in tobacco will increase the survival rate of transgenic plants under salt stress. *GarWRKY5* participates in the salt stress response in *G. aridum* through a signaling pathway mediated by jasmonic acid or salicylic acid, and overexpressed *GarWRKY5* in *Arabidopsis* enhances salt tolerance (Guo et al., 2019). It is worth noting that these genes or metabolism pathways were also identified in our research. The same salt stress response genes or metabolic pathways in callus suggest that calli might provide an effective screening tool that is relevant to salt stress in whole plants.

### Most Genes Related to ABA Metabolism or TFs Are Important to Salt Tolerance Both in Cotton Plants and in Callus

Abscisic acid is a key regulatory factor in response to salt stress in plants (Moons, 1997; Gong et al., 2020). *ABA2* and *NCED* are critical genes involved in the regulation of the abscisic acid biosynthesis pathway, and the *CYP707A* gene is a key enzyme for ABA catabolism. In maize plants, *ZmABA2* was upregulated in leaves after 2 h of 250 mM NaCl treatment; overexpression of *ZmABA2* in tobacco could increase ABA content and enhance the tolerance to salt stress (Ma et al., 2016). The expression abundance of *OsNCED5* was significantly induced in roots and leaves when the rice is under high-salt conditions; overexpressed *OsNCED5* in rice will increase the salt-tolerance (Huang et al., 2019). In addition, *OsNCED3* and *OsNCED4* had similar expression patterns with *OsNCED5* under salt stress conditions (Huang et al., 2019). In our study, through treatment with 200 mM NaCl in cotton callus, we also identified 28 differentially expressed genes involved in the ABA synthesis pathway under salt stress, including seven *ABA2* and two *NCED* genes upregulated under salt stress, and two *CYP707A* genes that were downregulated. These genes may respond to salt stress through the ABA pathway both in cotton plants and callus (Cheng et al., 2009; Barrero et al., 2010; Zheng et al., 2012).

When plants are subjected to salt stress, the content of ABA increases and binds to ABA receptor proteins (*PYR/PYL/RCAR*) to regulate the activity of *PP2C*. The activated *PP2C* genes directly interact with *SnRK2* and transmit ABA signaling to the downstream *AREB/ABF* transcription factors, further mediating the expression patterns of salt stress-responsive genes (Wei et al., 2017). The results of transcriptome analysis of roots and leaves in *G. klotzschianum* showed that the *PYL* gene is significantly downregulated, and the *PP2C* and *SnRK2* genes are significantly upregulated in salt stress (Wei et al., 2017), and similar results were demonstrated in our study in callus of cotton.

In our study, we identified only one *MKK2* (*MAPKK*) gene and it was upregulated under salt stress in *G. raimondii* callus, but there were two *MKK2*, one *MEKK1* (*MAPKKK*), and one *MPK4* (*MAPK*) that were induced and upregulated under salt stress in *G. sturtianum* callus. MAPK is a mitogen-activated protein kinase that interacts with ABA2 and regulates ABA synthesis (Verma et al., 2020). In general, MAPK activates downstream elements such as transcription factors and causes related physiological and biochemical reactions in response to stress. Most of the *MAPK* genes could respond to salt stress in different tissues of plants. *DSM1* (*MAPKKK*), *OsMAPK44*, and *OsMAPK5* were upregulated under salt stress; overexpression of *OsMAPK44* and *OsMAPK5* in rice could increase the salt tolerance effectively (Xiong and Yang, 2003; Jeong et al., 2006; Ning et al., 2010). The transcriptional level of *ZmMPK3* was significantly upregulated under high-salt conditions in maize seedlings (Wang et al., 2010). In addition, overexpression of *ZmMPK5* in tobacco increased resistance to salt-alkali stress through scavenging excess reactive oxygen species (Zhang D. et al., 2014). *GhMAPK, GhMPK2*, and *GhMPK4* play important roles in salt stress response in upland cotton (Wang et al., 2007, 2015; Zhang et al., 2011), and overexpression of *GhMAP3K40* increases tolerance to salt stress during the germination period in cotton (Chen et al., 2015).

Transcription factors are critical to transfer stress signaling into the tolerance response genes. Multiple TFs such as *bZIP, WRKY, ERF, MYB, bHLH*, and *NAC* families are differentially expressed under salt stress (Golldack et al., 2011). Large numbers of transcription factors were annotated among the DEGs shared by two cotton species under salt stress in callus, including *ERF, MYB, bHLH, NAC, bZIP, C2H2*, and *WRKY*. Among them, members of the *NAC* and *WRKY* families occupy a large proportion, and most of these were upregulated under salt stress, indicating that these two TF families may positively regulate salt stress response in callus. Additional transcription factors may be also important in maintaining salt stress response. Overexpression of the *GRAS* transcription factor improves salt tolerance in *Arabidopsis* and yeast (Shi et al., 2015; Yuan et al., 2016; Zhang et al., 2020). *OsDOF15* mediates ethylene biosynthesis in rice, and it is important in response to salt stress (Qin et al., 2019). Some *GhDof* genes are also differentially expressed in upland cotton under salt stress, and overexpression of *GhDof1* in cotton increases salt tolerance compared to wild-type plants (Guo et al., 2015; Shi et al., 2015; Su et al., 2017a). *WOX* is an important transcriptional regulator involved in the development of plant embryos and is differentially expressed in abiotic stress conditions such as drought, salt, and cold. *OsWOX3* and *OsWOX5* are rapidly upregulated after treatment with 150 mM NaCl in rice (Cheng et al., 2014). We also identified a certain number of transcription factors such as *Dof*, *GRAS*, and *WOX* in callus under salt stress, suggesting that these TFs may also be important to salt tolerance in wild cotton not only in whole plants but also in callus.

## Conclusion

**Figure 6:**
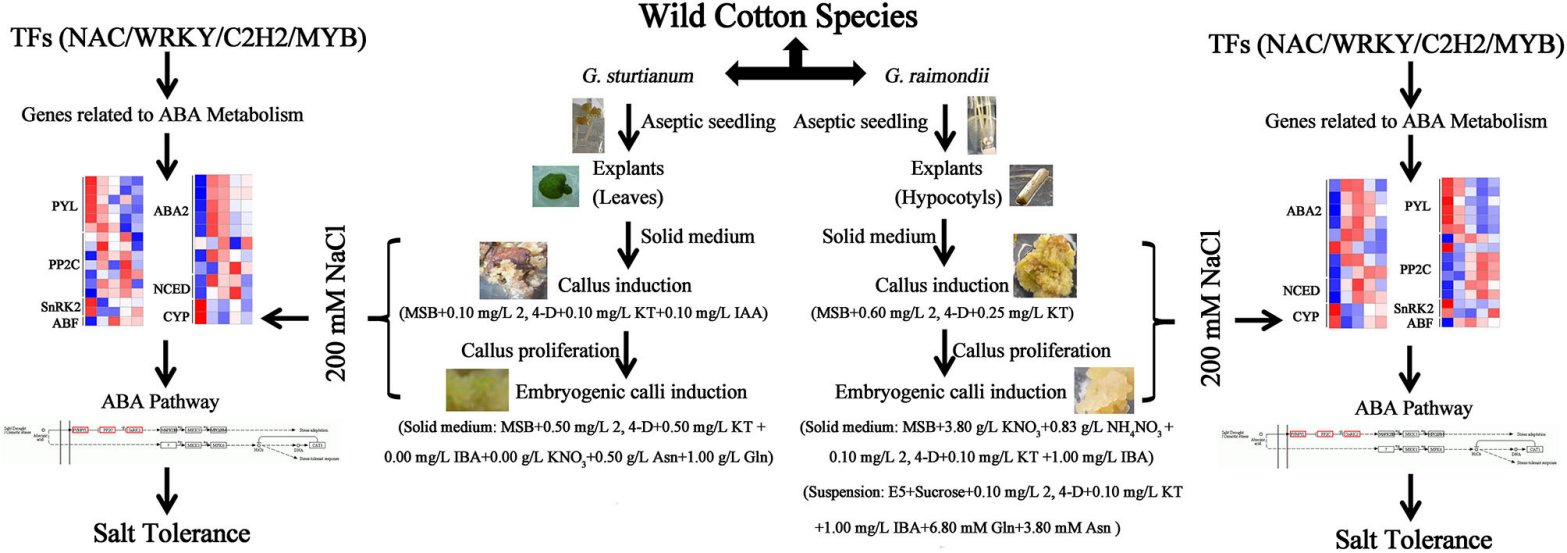
The mode of embryogenic callus induction and salt tolerance of two wild cotton species *G. sturtianum* and *G. raimondii*.

Here, we demonstrate the successful induction of embryogenic callus from two wild diploid cotton species. We used these to perform the first comprehensive transcriptomic analysis of callus under salt stress, revealing numerous differentially expressed genes including TF families related to ABA biosynthesis and signal transduction, such as *SnRK2, ABA2, NAC*, and *WRKY* (Figure 6). Our study provides a foundation for plant regeneration through protoplast fusion and a useful resource for improving agronomically relevant salt resistance using beneficial genes from wild cotton species.

## Data Availability Statement

The datasets presented in this study can be found in online repositories. The names of the repository/repositories and accession number(s) can be found below: NCBI SRA BioProject, accession no: PRJNA736855.

## Author Contributions

HN, YW, and CW conducted the experiments and analyzed the data. HN prepared the manuscript. YS attended benchwork. CG and JW attended the discussion and revised the manuscript. JH conceived the experiments, provided the experimental platform, and revised the manuscript. All authors approved the final manuscript.

## Conflict of Interest

The authors declare that the research was conducted in the absence of any commercial or financial relationships that could be construed as a potential conflict of interest.

## Publisher's Note

All claims expressed in this article are solely those of the authors and do not necessarily represent those of their affiliated organizations, or those of the publisher, the editors and the reviewers. Any product that may be evaluated in this article, or claim that may be made by its manufacturer, is not guaranteed or endorsed by the publisher.

## Supplementary Material

Supplementary Figure 1 Correlation and different expression genes analysis of RNA-seq. **(A)** Statistical analysis of correlation coefficient between each sample; **(B)** principal component analysis of each sample; **(C)** statistics of the number of DEGs in *G. raimondii* and *G. sturtianum*; **(D,E)** Venn diagrams of DEGs in different salt stress stages of *G. raimondii* **(D)** and *G. sturtianum* **(E)**.

Supplementary Figure 2 Callus suspension culture of *G. raimondii* and *G. sturtianum*. **(A)** Callus suspension culture of *G. raimondii*, **(B)** the callus of *G. raimondii* obtained from the suspension culture was inoculated to the solid medium, **(C)** callus suspension culture of *G. sturtianum*, **(D)** the callus of *G. sturtianum* obtained from the suspension culture was inoculated on solid medium.

Supplementary Figure 3 Identification and functional analysis of different expression genes during diverse stages under salt stress in *G. raimondii* and *G. sturtianum*; **(A)** The heatmap of co-genes which different expressed during 4 stages under salt stress in *G. raimondii*; **(B)** qRT-PCR results of 14 genes under salt stress in *G. raimondii* and *G. sturtianum*; **(C)** GO enrichment analysis of common DEGs in *G. raimondii* and *G. sturtianum*. *GHUBQ7* gene as an internal control, and the error bars indicated the standard error of the means.

Supplementary Figure 4 MAPK signaling pathway.

Supplementary Table 1 The culture media for embryogenic callus induction of *G. sturtianum*.

Supplementary Table 2 Media used in embryogenic callus induction of *G. raimondii*.

Supplementary Table 3 The medium components for callus suspension culture of *G. raimondii* and *G. sturtianum*.

Supplementary Table 4 Summary of RNA-Seq data quality in different libraries.

Supplementary Table 5 Alignment result of sample sequencing data to the reference genome.

Supplementary Table 6 The comparison of nitrogen source used in embryonic callus of *G. sturtianum*.

Supplementary Table 7 The comparison of FeSO_4_, CuSO_4_ used in embryonic callus induction of *G. sturtianum*.

Supplementary Table 8 Primers of target genes for qRT-PCR.

Supplementary Table 9 List of genes related to MAPK signaling pathway.

## Abbreviations

2, 4-D: 2, 4-Dichlorophenoxyacetic acid
ABA: Abscisic acid
*ABA2*: Xanthoxin dehydrogenase genes
BBM: Baby boom
CTAB: Cetyltrimethyl ammonium bromide
DEGs: Differentially expressed genes
FC: Fold change
FDR: False discovery rate
GO: Gene ontology
IAA: Indole-3-acetic acid
IBI: Indole-3-butyric acid
KT: Kinetin
KEGG: Kyoto Encyclopedia of Genes and Genomes
LEC: Leafy cotyledon
MSB_5_: MS salts and B_5_ vitamins
*NAC*: *NAM* (no apical meristem), *ATAT1*/*2*, and *CUC2* (cup-shaped cotyledon)
PCA: Principal component analysis
PGRs: Plant growth regulators
qRT-PCR: Quantitative real-time PCR
TFs: Transcription factors
*SnRK*: Sucrose non-fermenting 1-related protein kinases
SE: Somatic embryogenesis
*WRKY*: WRKY class of zinc-finger proteins.
