# Supplementary figures and images for: Embryogenic Calli Induction and Salt Stress Response Revealed by RNA-Seq in Diploid Wild Species Gossypium sturtianum and Gossypium raimondii
Source: Front Plant Sci. 2021 Aug 25;12:715041. doi: 10.3389/fpls.2021.715041 (PMC8424188; doi:10.3389/fpls.2021.715041)

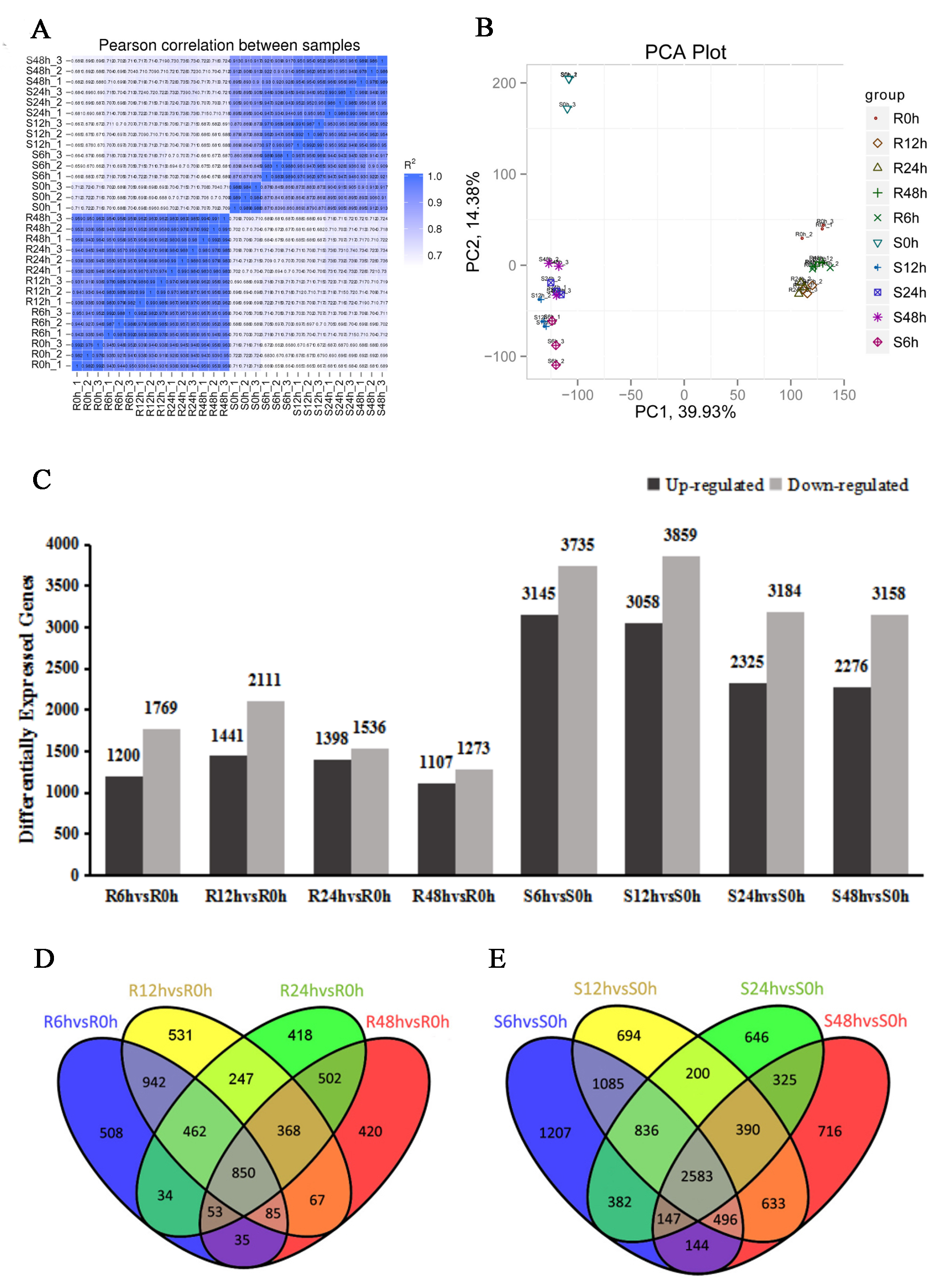

Supplement: Supplementary Figure 1 — Correlation and different expression genes analysis of RNA-seq. (A) Statistical analysis of correlation coefficient between each sample; (B) principal component analysis of each sample; (C) statistics of the number of DEGs in G. raimondii and G. sturtianum; (D,E) Venn diagrams of DEGs in different salt stress stages of G. raimondii (D) and G. sturtianum (E). [file Data_Sheet_1.zip › 20210809-Additional files/Figure. S1.jpg]

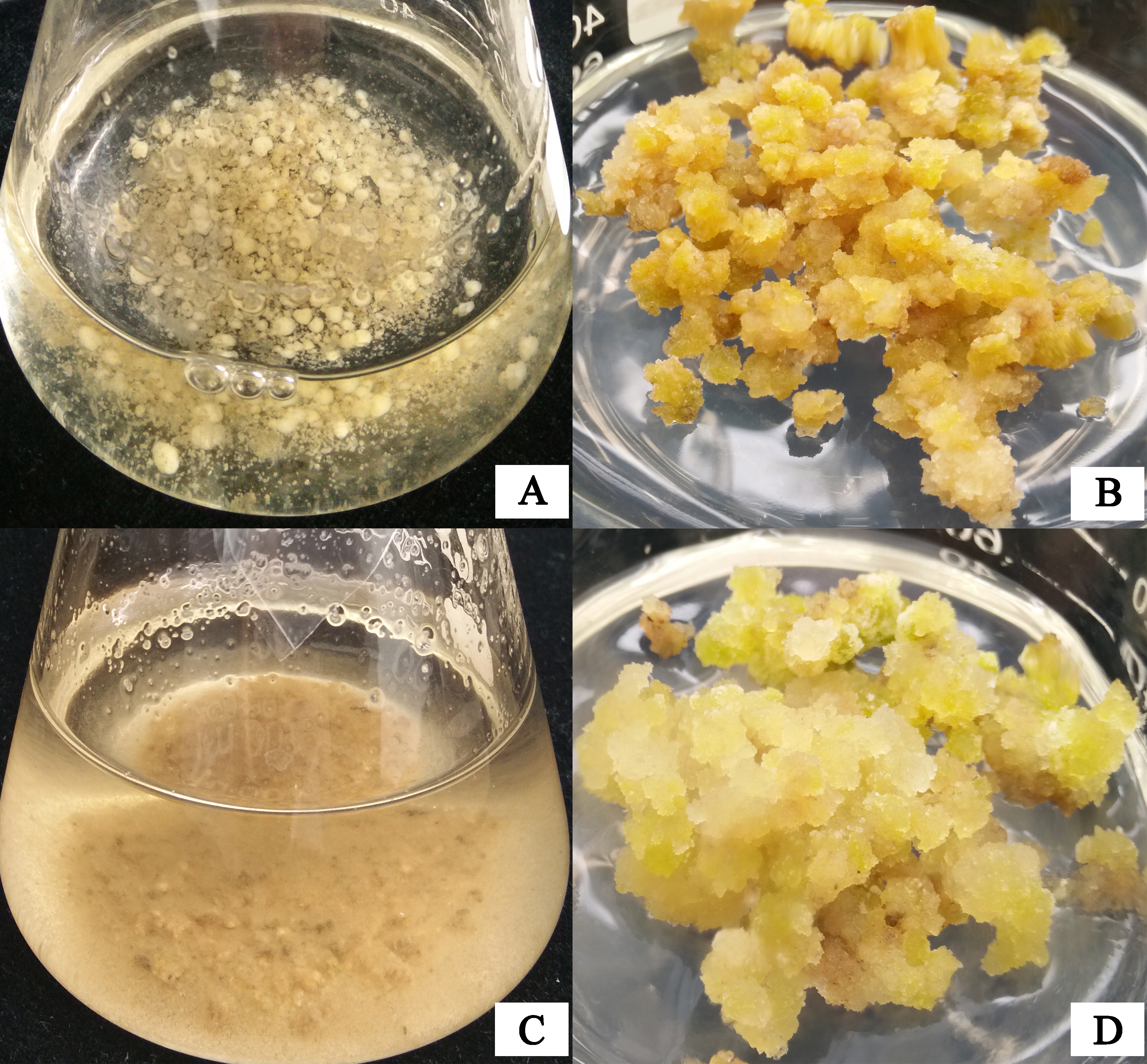

Supplement: Supplementary Figure 1 — Correlation and different expression genes analysis of RNA-seq. (A) Statistical analysis of correlation coefficient between each sample; (B) principal component analysis of each sample; (C) statistics of the number of DEGs in G. raimondii and G. sturtianum; (D,E) Venn diagrams of DEGs in different salt stress stages of G. raimondii (D) and G. sturtianum (E). [file Data_Sheet_1.zip › 20210809-Additional files/Figure. S2.jpg]

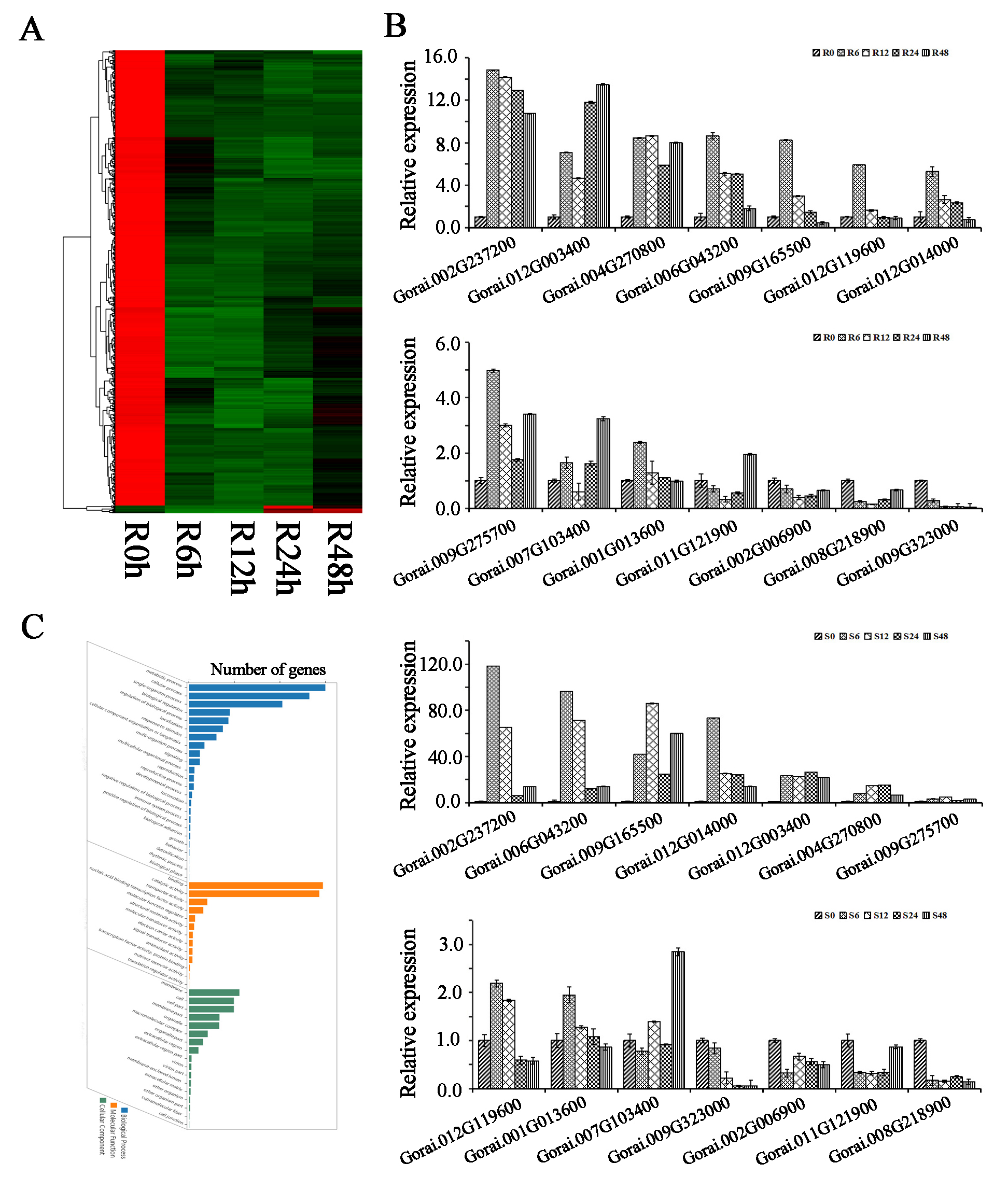

Supplement: Supplementary Figure 1 — Correlation and different expression genes analysis of RNA-seq. (A) Statistical analysis of correlation coefficient between each sample; (B) principal component analysis of each sample; (C) statistics of the number of DEGs in G. raimondii and G. sturtianum; (D,E) Venn diagrams of DEGs in different salt stress stages of G. raimondii (D) and G. sturtianum (E). [file Data_Sheet_1.zip › 20210809-Additional files/Figure. S3.jpg]

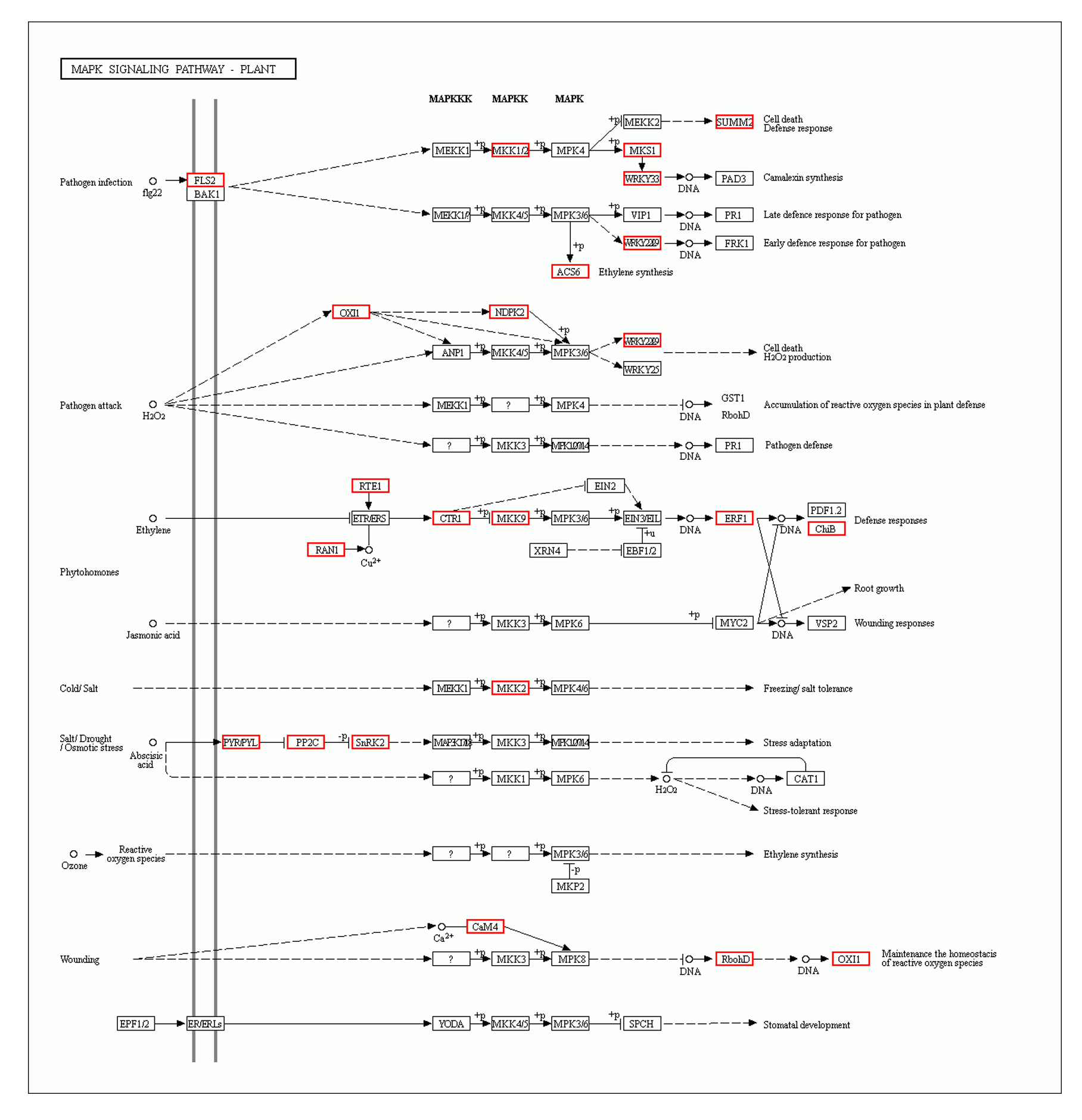

Supplement: Supplementary Figure 1 — Correlation and different expression genes analysis of RNA-seq. (A) Statistical analysis of correlation coefficient between each sample; (B) principal component analysis of each sample; (C) statistics of the number of DEGs in G. raimondii and G. sturtianum; (D,E) Venn diagrams of DEGs in different salt stress stages of G. raimondii (D) and G. sturtianum (E). [file Data_Sheet_1.zip › 20210809-Additional files/Figure. S4.jpg]
