# Supplementary material for: Embryogenic Calli Induction and Salt Stress Response Revealed by RNA-Seq in Diploid Wild Species Gossypium sturtianum and Gossypium raimondii
Source: Front Plant Sci. 2021 Aug 25;12:715041. doi: 10.3389/fpls.2021.715041 (PMC8424188; doi:10.3389/fpls.2021.715041)
Supplement: Supplementary Figure 1 — Correlation and different expression genes analysis of RNA-seq. (A) Statistical analysis of correlation coefficient between each sample; (B) principal component analysis of each sample; (C) statistics of the number of DEGs in G. raimondii and G. sturtianum; (D,E) Venn diagrams of DEGs in different salt stress stages of G. raimondii (D) and G. sturtianum (E). [file Data_Sheet_1.zip › 20210809-Additional files/Table S1.doc]

**Table S1** The culture media for embryogenic callus induction of *G. sturtianum*

| Medium | | Hormone combination (mg·L^-1^) | |
| --- | --- | --- | --- |
|  |  | 2, 4-D | KT |
| Nitrogen source deficiency medium | N1 | 0.50 | 0.50 |
|  | N2 | 0.50 | 0.25 |
|  | N3 | 1.00 | 0.50 |
| Carbon source deficiency medium | C1 | 0.50 | 0.50 |
|  | C2 | 0.50 | 0.25 |
|  | C3 | 1.00 | 0.50 |

| Medium | Nutrients (g·L^-1^) | | | | Hormone combination (mg·L^-1^) | | | |
| --- | --- | --- | --- | --- | --- | --- | --- | --- |
|  | KNO_3_ | MgSO_4_ | Gln | Asn | IAA | IBA | 2, 4-D | KT |
| N4 | 0.00 | 0.00 | 1.00 | 0.50 | 0.00 | 0.00 | 0.50 | 0.50 |
| N5 | 0.00 | 0.00 | 1.00 | 0.50 | 1.00 | 0.00 | 0.50 | 0.50 |
| N6 | 0.00 | 0.00 | 1.00 | 0.50 | 0.00 | 0.00 | 1.00 | 0.50 |
| N7 | 0.00 | 0.00 | 1.00 | 0.50 | 1.00 | 0.00 | 1.00 | 0.50 |

**Notes:** nitrogen source: KNO_3_; carbon source: sucrose.
