# Supplementary material for: Embryogenic Calli Induction and Salt Stress Response Revealed by RNA-Seq in Diploid Wild Species Gossypium sturtianum and Gossypium raimondii
Source: Front Plant Sci. 2021 Aug 25;12:715041. doi: 10.3389/fpls.2021.715041 (PMC8424188; doi:10.3389/fpls.2021.715041)
Supplement: Supplementary Figure 1 — Correlation and different expression genes analysis of RNA-seq. (A) Statistical analysis of correlation coefficient between each sample; (B) principal component analysis of each sample; (C) statistics of the number of DEGs in G. raimondii and G. sturtianum; (D,E) Venn diagrams of DEGs in different salt stress stages of G. raimondii (D) and G. sturtianum (E). [file Data_Sheet_1.zip › 20210809-Additional files/Table S2.doc]

**Table S2** Media used in embryogenic callus induction of *G. raimondii*

| Medium | Nitrogen source（g·L^-1^） | Ratio of hormomes (mg·L^-1^) | | | Carbon source | Color, texture and proliferation rate of calli | Embryogenic  calli obtained |
| --- | --- | --- | --- | --- | --- | --- | --- |
|  |  | 2, 4-D KT IBA | | |  |  |  |
| RE1 | 3.80KNO_3_ | - | 0.10 | 0.25 | Glucose | Yellow-green, loose, moderate | No |
| RE2 | 3.80KNO_3_ | 0.05 | 0.20 | 0.50 | Sucrose | Brown, died, slow | No |
| RE3 | 3.80KNO_3_ | 0.10 | 0.30 | 1.00 | 1/2 Glucose +1/2 Sucrose | Yellow-green, loose, moderate | No |
| RE4 | 1.90KNO_3_+1.65NH_4_NO_3_ | - | 0.10 | 0.50 | Sucrose | Yellow-white, loose, slow | No |
| RE5 | 1.90KNO_3_+1.65NH_4_NO_3_ | 0.05 | 0.20 | 1.00 | 1/2 Glucose +1/2 Sucrose | Yellow-green, hard, moderate | No |
| RE6 | 1.90KNO_3_+1.65NH_4_NO_3_ | 0.10 | 0.30 | 0.25 | Glucose | Green, loose, moderate | No |
| RE7 | 3.80KNO_3_+0.83NH_4_NO_3_ | - | 0.20 | 0.25 | 1/2 Glucose +1/2 Sucrose | Yellow-white, loose, moderate | No |
| RE8 | 3.80KNO_3_+0.83NH_4_NO_3_ | 0.05 | 0.30 | 0.50 | Glucose | Yellow-green, loose, moderate | No |
| RE9 | 3.80KNO_3_+0.83NH_4_NO_3_ | 0.10 | 0.10 | 1.00 | Sucrose | Yellow-green, loose, moderate, browning | Yes |
| RE10 | 3.80KNO_3_ | - | 0.30 | 1.00 | Sucrose | Green, loose, moderate | No |
| RE11 | 3.80KNO_3_ | 0.05 | 0.10 | 0.25 | 1/2 Glucose +1/2 Sucrose | Yellow-green, hard, moderate | Yes |
| RE12 | 3.80KNO_3_ | 0.10 | 0.20 | 0.50 | Glucose | Yellow-green, hard, extremely slow | No |
| RE13 | 1.90KNO_3_+1.65NH_4_NO_3_ | - | 0.20 | 1.00 | Glucose | Yellow-white, died, slow | No |
| RE14 | 1.90KNO_3_+1.65NH_4_NO_3_ | 0.05 | 0.30 | 0.25 | Sucrose | Yellow-brown, loose, slow | Yes |
| RE15 | 1.90KNO_3_+1.65NH_4_NO_3_ | 0.10 | 0.10 | 0.50 | 1/2 Glucose +1/2 Sucrose | Yellow-green, hard, moderate | No |
| RE16 | 3.80KNO_3_+0.83NH_4_NO_3_ | - | 0.30 | 0.50 | 1/2 Glucose +1/2 Sucrose | Yellow-green, hard, moderate | No |
| RE17 | 3.80KNO_3_+0.83NH_4_NO_3_ | 0.05 | 0.10 | 1.00 | Glucose | Yellow-white, loose, slow | No |
