# Supplementary material for: Embryogenic Calli Induction and Salt Stress Response Revealed by RNA-Seq in Diploid Wild Species Gossypium sturtianum and Gossypium raimondii
Source: Front Plant Sci. 2021 Aug 25;12:715041. doi: 10.3389/fpls.2021.715041 (PMC8424188; doi:10.3389/fpls.2021.715041)
Supplement: Supplementary Figure 1 — Correlation and different expression genes analysis of RNA-seq. (A) Statistical analysis of correlation coefficient between each sample; (B) principal component analysis of each sample; (C) statistics of the number of DEGs in G. raimondii and G. sturtianum; (D,E) Venn diagrams of DEGs in different salt stress stages of G. raimondii (D) and G. sturtianum (E). [file Data_Sheet_1.zip › 20210809-Additional files/Table S3.doc]

***Table S3*** *The medium components for callus suspension culture of* *G. raimondii and G. sturtianum*

| Medium | Basic medium ingredients | 2,4-D  (mg·L^-1^) | KT  (mg·L^-1^) | IAA  (mg·L^-1^) | IBA  (mg·L^-1^) | KNO_3_  (g·L^-1^) | Amino acid |
| --- | --- | --- | --- | --- | --- | --- | --- |
| REL | E5+3%（W/V）sucrose | 0.10 | 0.10 | - | 1.00 | - | 6.80 mM Gln+  3.80 mM Asn |
| SEL | MSN+3%（W/V）glucose | 0.20 | - | 0.95 | 0.30 | 0.10 | 6.80 mM Gln+  3.80 mM Asn |

Notes: REL: liquid medium of *G. raimondii*, SEL: liquid medium of *G. sturtianum*
