# Supplementary material for: Embryogenic Calli Induction and Salt Stress Response Revealed by RNA-Seq in Diploid Wild Species Gossypium sturtianum and Gossypium raimondii
Source: Front Plant Sci. 2021 Aug 25;12:715041. doi: 10.3389/fpls.2021.715041 (PMC8424188; doi:10.3389/fpls.2021.715041)
Supplement: Supplementary Figure 1 — Correlation and different expression genes analysis of RNA-seq. (A) Statistical analysis of correlation coefficient between each sample; (B) principal component analysis of each sample; (C) statistics of the number of DEGs in G. raimondii and G. sturtianum; (D,E) Venn diagrams of DEGs in different salt stress stages of G. raimondii (D) and G. sturtianum (E). [file Data_Sheet_1.zip › 20210809-Additional files/Table S4.doc]

**Table S4** Summary of RNA-Seq data quality in different libraries

| Sample name | Raw reads | Clean reads | Clean bases | Error rate (%) | Q20 (%) | Q30 (%) | GC content (%) |
| --- | --- | --- | --- | --- | --- | --- | --- |
| R0h_1 | 59,533,216 | 58,552,314 | 8.78G | 0.03 | 97.89 | 94.00 | 44.42 |
| R0h_2 | 63,762,026 | 62,711,496 | 9.41G | 0.03 | 97.71 | 93.54 | 44.32 |
| R0h_3 | 57,629,962 | 56,645,426 | 8.50G | 0.03 | 97.93 | 94.13 | 44.48 |
| R6h_1 | 59,338,730 | 58,366,106 | 8.75G | 0.03 | 97.95 | 94.08 | 44.44 |
| R6h_2 | 60,622,636 | 59,575,352 | 8.94G | 0.02 | 98.04 | 94.27 | 44.23 |
| R6h_3 | 53,128,178 | 52,172,698 | 7.83G | 0.03 | 97.96 | 94.14 | 44.46 |
| R12h_1 | 57,269,460 | 56,244,542 | 8.44G | 0.03 | 97.85 | 93.83 | 44.33 |
| R12h_2 | 64,048,662 | 62,929,382 | 9.44G | 0.02 | 98.03 | 94.28 | 44.27 |
| R12h_3 | 69,008,902 | 67,746,058 | 10.16G | 0.03 | 97.93 | 94.03 | 44.32 |
| R24h_1 | 57,905,920 | 56,908,792 | 8.54G | 0.03 | 97.93 | 94.05 | 44.41 |
| R24h_2 | 49,751,100 | 48,843,846 | 7.33G | 0.03 | 98.00 | 94.20 | 44.48 |
| R24h_3 | 59,942,328 | 58,609,832 | 8.79G | 0.03 | 98.01 | 94.23 | 44.45 |
| R48h_1 | 67,362,064 | 65,997,098 | 9.90G | 0.03 | 97.92 | 94.05 | 44.32 |
| R48h_2 | 51,638,314 | 50,766,926 | 7.62G | 0.03 | 97.99 | 94.11 | 44.28 |
| R48h_3 | 68428116 | 67121542 | 10.07G | 0.03 | 97.94 | 94.10 | 44.43 |
| S0h_1 | 55,021,482 | 53,831,060 | 8.07G | 0.03 | 97.91 | 94.02 | 43.97 |
| S0h_2 | 51,379,020 | 50,606,172 | 7.59G | 0.03 | 97.74 | 93.60 | 44.13 |
| S0h_3 | 48,516,040 | 47,922,942 | 7.19G | 0.03 | 97.73 | 93.60 | 44.11 |
| S6h_1 | 46,030,900 | 45,540,968 | 6.83G | 0.03 | 97.87 | 93.84 | 44.01 |
| S6h_2 | 54,667,554 | 54,018,874 | 8.10G | 0.03 | 97.84 | 93.82 | 44.05 |
| S6h_3 | 49,493,142 | 48,834,106 | 7.33G | 0.03 | 97.68 | 93.41 | 44.08 |
| S12h_1 | 54,646,944 | 54,053,164 | 8.11G | 0.03 | 97.73 | 93.53 | 44.09 |
| S12h_2 | 54,896,144 | 54,205,870 | 8.13G | 0.03 | 97.81 | 93.77 | 44.02 |
| S12h_3 | 48,407,052 | 47,778,392 | 7.17G | 0.03 | 97.75 | 93.63 | 44.25 |
| S24h_1 | 46,694,088 | 46,166,662 | 6.92G | 0.03 | 97.80 | 93.73 | 44.27 |
| S24h_2 | 42,756,760 | 42,289,544 | 6.34G | 0.03 | 97.86 | 93.81 | 44.16 |
| S24h_3 | 46,871,724 | 46,323,860 | 6.95G | 0.03 | 97.92 | 93.98 | 44.24 |
| S48h_1 | 44,528,288 | 43,890,298 | 6.58G | 0.03 | 97.78 | 93.69 | 44.41 |
| S48h_2 | 50,081,402 | 49,248,696 | 7.39G | 0.03 | 97.73 | 93.59 | 44.25 |
| S48h_3 | 64,303,778 | 63,311,352 | 9.50G | 0.03 | 97.81 | 93.81 | 44.30 |
