# Supplementary material for: Embryogenic Calli Induction and Salt Stress Response Revealed by RNA-Seq in Diploid Wild Species Gossypium sturtianum and Gossypium raimondii
Source: Front Plant Sci. 2021 Aug 25;12:715041. doi: 10.3389/fpls.2021.715041 (PMC8424188; doi:10.3389/fpls.2021.715041)
Supplement: Supplementary Figure 1 — Correlation and different expression genes analysis of RNA-seq. (A) Statistical analysis of correlation coefficient between each sample; (B) principal component analysis of each sample; (C) statistics of the number of DEGs in G. raimondii and G. sturtianum; (D,E) Venn diagrams of DEGs in different salt stress stages of G. raimondii (D) and G. sturtianum (E). [file Data_Sheet_1.zip › 20210809-Additional files/Table S5.doc]

**Table S5** Alignment result of sample sequencing data to the reference genome

| Sample name | Total reads | Total mapped (%) | Multiple mapped (%) | Uniquely mapped (%) | Read-1 (%) | Read-2 (%) | Reads map to '+' (%) | Reads map to '-' (%) | Non-splice reads (%) | Splice reads (%) |
| --- | --- | --- | --- | --- | --- | --- | --- | --- | --- | --- |
| R0h_1 | 58,552,314 | 96.63 | 2.92 | 93.72 | 47.00 | 46.71 | 46.83 | 46.89 | 60.07 | 33.65 |
| R0h_2 | 62,711,496 | 96.88 | 2.83 | 94.05 | 47.18 | 46.87 | 46.99 | 47.06 | 61.05 | 33.00 |
| R0h_3 | 56,645,426 | 96.91 | 3.02 | 93.88 | 47.05 | 46.83 | 46.90 | 46.98 | 59.10 | 34.79 |
| R6h_1 | 58,366,106 | 96.93 | 3.18 | 93.75 | 46.96 | 46.79 | 46.82 | 46.93 | 57.81 | 35.94 |
| R6h_2 | 59,575,352 | 97.11 | 3.07 | 94.04 | 47.11 | 46.94 | 46.99 | 47.06 | 58.60 | 35.45 |
| R6h_3 | 52,172,698 | 97.03 | 3.32 | 93.72 | 46.97 | 46.74 | 46.82 | 46.90 | 58.15 | 35.57 |
| R12h_1 | 56,244,542 | 96.97 | 3.33 | 93.64 | 46.96 | 46.69 | 46.77 | 46.87 | 58.28 | 35.36 |
| R12h_2 | 62,929,382 | 97.38 | 3.21 | 94.17 | 47.16 | 47.01 | 47.04 | 47.13 | 58.62 | 35.55 |
| R12h_3 | 67,746,058 | 97.06 | 3.36 | 93.70 | 46.96 | 46.75 | 46.80 | 46.91 | 58.28 | 35.42 |
| R24h_1 | 56,908,792 | 97.17 | 2.79 | 94.39 | 47.32 | 47.07 | 47.15 | 47.24 | 58.69 | 35.70 |
| R24h_2 | 48,843,846 | 97.03 | 2.74 | 94.29 | 47.24 | 47.05 | 47.10 | 47.19 | 58.78 | 35.51 |
| R24h_3 | 58,609,832 | 96.97 | 3.01 | 93.97 | 47.05 | 46.91 | 46.94 | 47.03 | 59.03 | 34.93 |
| R48h_1 | 65,997,098 | 96.83 | 2.92 | 93.91 | 47.05 | 46.86 | 46.91 | 47.00 | 59.34 | 34.57 |
| R48h_2 | 50,766,926 | 97.14 | 2.48 | 94.66 | 47.41 | 47.25 | 47.31 | 47.35 | 59.44 | 35.23 |
| R48h_3 | 67,121,542 | 96.91 | 2.95 | 93.96 | 47.07 | 46.89 | 46.94 | 47.02 | 59.10 | 34.85 |
| S0h_1 | 53,831,060 | 78.87 | 2.32 | 76.54 | 38.40 | 38.15 | 38.27 | 38.28 | 45.05 | 31.50 |
| S0h_2 | 50,606,172 | 78.42 | 2.49 | 75.93 | 38.04 | 37.89 | 37.97 | 37.96 | 44.64 | 31.29 |
| S0h_3 | 47,922,942 | 79.25 | 2.27 | 76.98 | 38.56 | 38.42 | 38.49 | 38.49 | 45.68 | 31.30 |
| S6h_1 | 45,540,968 | 81.95 | 3.27 | 78.68 | 39.40 | 39.29 | 39.38 | 39.30 | 45.66 | 33.03 |
| S6h_2 | 54,018,874 | 81.31 | 3.57 | 77.74 | 38.92 | 38.82 | 38.87 | 38.87 | 45.44 | 32.30 |
| S6h_3 | 48,834,106 | 82.01 | 3.49 | 78.52 | 39.37 | 39.15 | 39.26 | 39.26 | 45.51 | 33.01 |
| S12h_1 | 54,053,164 | 82.73 | 3.20 | 79.52 | 39.86 | 39.67 | 39.82 | 39.71 | 45.97 | 33.55 |
| S12h_2 | 54,205,870 | 82.45 | 3.12 | 79.33 | 39.75 | 39.58 | 39.72 | 39.62 | 45.93 | 33.41 |
| S12h_3 | 47,778,392 | 82.20 | 3.36 | 78.85 | 39.50 | 39.35 | 39.46 | 39.39 | 45.73 | 33.11 |
| S24h_1 | 46,166,662 | 82.86 | 2.68 | 80.18 | 40.16 | 40.02 | 40.11 | 40.08 | 46.53 | 33.66 |
| S24h_2 | 42,289,544 | 82.91 | 2.49 | 80.42 | 40.25 | 40.16 | 40.25 | 40.17 | 47.15 | 33.27 |
| S24h_3 | 46,323,860 | 82.71 | 2.80 | 79.91 | 39.97 | 39.93 | 39.98 | 39.93 | 46.43 | 33.48 |
| S48h_1 | 43,890,298 | 82.74 | 3.13 | 79.61 | 39.85 | 39.76 | 39.88 | 39.72 | 45.79 | 33.81 |
| S48h_2 | 49,248,696 | 82.19 | 3.03 | 79.16 | 39.66 | 39.50 | 39.67 | 39.49 | 45.73 | 33.43 |
| S48h_3 | 63,311,352 | 82.00 | 3.06 | 78.94 | 39.51 | 39.43 | 39.56 | 39.37 | 45.58 | 33.36 |
