# Supplementary material for: Embryogenic Calli Induction and Salt Stress Response Revealed by RNA-Seq in Diploid Wild Species Gossypium sturtianum and Gossypium raimondii
Source: Front Plant Sci. 2021 Aug 25;12:715041. doi: 10.3389/fpls.2021.715041 (PMC8424188; doi:10.3389/fpls.2021.715041)
Supplement: Supplementary Figure 1 — Correlation and different expression genes analysis of RNA-seq. (A) Statistical analysis of correlation coefficient between each sample; (B) principal component analysis of each sample; (C) statistics of the number of DEGs in G. raimondii and G. sturtianum; (D,E) Venn diagrams of DEGs in different salt stress stages of G. raimondii (D) and G. sturtianum (E). [file Data_Sheet_1.zip › 20210809-Additional files/Table S6.doc]

**Table S6** The comparison of nitrogen source used in embryonic callus of *G. sturtianum*

| Medium | Hormone combination (mg·L^-1^） | KNO_3_ (g·L^-1^） | Callus description |
| --- | --- | --- | --- |
| C1 | （0.20）2,4-D+（0.10）KT | 3.80 | Gray, nonembryogenic |
| C2 | （0.20）2,4-D+（0.10）KT | 1.90 | Gray, embryogenic |
| C3 | （0.20）2,4-D+（0.10）KT | 7.60 | Died |
| E1 | （0.40）2,4-D+（0.20）KT | 3.80 | Yellow, nonembryogenic |
| E2 | （0.40）2,4-D+（0.20）KT | 1.90 | nonembryogenic |
| E3 | （0.40）2,4-D+（0.20）KT | 7.60 | Yellow, nonembryogenic |
| EI8 | （0.20）2,4-D+（0.10）KT+（0.50）IBA | 3.80 | Gray, nonembryogenic |
| EI9 | （0.20）2,4-D+（0.10）KT+（0.50）IBA | 7.60 | Yellow, embryogenic |
