# Supplementary material for: Embryogenic Calli Induction and Salt Stress Response Revealed by RNA-Seq in Diploid Wild Species Gossypium sturtianum and Gossypium raimondii
Source: Front Plant Sci. 2021 Aug 25;12:715041. doi: 10.3389/fpls.2021.715041 (PMC8424188; doi:10.3389/fpls.2021.715041)
Supplement: Supplementary Figure 1 — Correlation and different expression genes analysis of RNA-seq. (A) Statistical analysis of correlation coefficient between each sample; (B) principal component analysis of each sample; (C) statistics of the number of DEGs in G. raimondii and G. sturtianum; (D,E) Venn diagrams of DEGs in different salt stress stages of G. raimondii (D) and G. sturtianum (E). [file Data_Sheet_1.zip › 20210809-Additional files/Table S7.doc]

**Table S7** The comparison of FeSO_4_, CuSO_4_ used in embryonic callus induction of *G. sturtianum*

| Medium | Hormone combination (mg·L^-1^） | KNO_3_ (g·L^-1^） | FeSO_4_ | CuSO_4_ | Callus description |
| --- | --- | --- | --- | --- | --- |
| C1 | （0.20）2,4-D+（0.10）KT | 3.80 | 1× | 1× | Yellow, fast proliferation, embryogenic |
| E4 | （0.40）2,4-D+（0.20）KT | 3.80 | 2× | 1× | Yellow, fast proliferation, embryogenic |
| E5 | （0.40）2,4-D+（0.20）KT | 3.80 | 3× | 1× | Sallow, brown, embryogenic |
| E6 | （0.20）2,4-D+（0.50）KT | 3.80 | 0.1 × | 1× | Died |
| E7 | （0.20）KT+（0.50）IBA | 1.90 | 1× | 5× | Died |
| E8 | （0.20）2,4-D+（0.20）KT+（0.50）IBA | 1.90 | 1× | 5× | Brown, slow proliferation, nonembryogenic |
| E9 | （0.20）KT+（0.50）IBA | 1.90 | 1× | - | Died |
| E10 | （0.20）2,4-D+（0.20）KT+（0.50）IBA | 1.90 | 2× | - | Fast proliferation, nonembryogenic |
