# Supplementary material for: Embryogenic Calli Induction and Salt Stress Response Revealed by RNA-Seq in Diploid Wild Species Gossypium sturtianum and Gossypium raimondii
Source: Front Plant Sci. 2021 Aug 25;12:715041. doi: 10.3389/fpls.2021.715041 (PMC8424188; doi:10.3389/fpls.2021.715041)
Supplement: Supplementary Figure 1 — Correlation and different expression genes analysis of RNA-seq. (A) Statistical analysis of correlation coefficient between each sample; (B) principal component analysis of each sample; (C) statistics of the number of DEGs in G. raimondii and G. sturtianum; (D,E) Venn diagrams of DEGs in different salt stress stages of G. raimondii (D) and G. sturtianum (E). [file Data_Sheet_1.zip › 20210809-Additional files/Table S8.doc]

**Table S8** Primers of target genes for qRT-PCR

| Primers | Sequence (5'-3') |
| --- | --- |
| Gorai.001G013600-F | ACACAAAGTGGTACGTTCAAAG |
| Gorai.001G013600-R | AATTAGCGAAGGAAGTTCATGC |
| Gorai.002G006900-F | CTTTGAGTGAAATGGATCGACC |
| Gorai.002G006900-R | ATCTCTCATCAACCTAGCAACC |
| Gorai.002G237200-F | GAGTTAACTGCATTTCGCCTAG |
| Gorai.002G237200-R | CCTCATCACTTGCCAAATACAG |
| Gorai.004G270800-F | ATGCATGTATATACTGTCCCCG |
| Gorai.004G270800-R | CCAAAAGCTACAAACTGGTCTC |
| Gorai.006G043200-F | AAAACTGCTTCATCCAGAAACC |
| Gorai.006G043200-R | AGCTTCTTGGGAATTTGTTGTC |
| Gorai.007G103400-F | TGACATGGTCACTGGTAATTGT |
| Gorai.007G103400-R | ATCAAAAGCAATGTTCCAGGAC |
| Gorai.008G218900-F | GTGCTCGTTACCAAATCTCATC |
| Gorai.008G218900-R | GAGAACAAGCTTCCTCAACTTG |
| Gorai.009G165500-F | CCAAGGCTTTACTCAATTTCCC |
| Gorai.009G165500-R | CTTTTTGAGCTTGAATTTGGCC |
| Gorai.009G275700-F | GGAGAACCAAGAATTGCAGAAG |
| Gorai.009G275700-R | CTTTTGATTCACCAAGGACCTG |
| Gorai.009G323000-F | GTCGTTGAATCATTCGTCGTAG |
| Gorai.009G323000-R | AAGATTGAAGGTTGCAACGTAC |
| Gorai.011G121900-F | CGTTTGAAAATCTGCGATTTCG |
| Gorai.011G121900-R | AGCTCCCACCAACATAACATAA |
| Gorai.012G003400-F | ACTTTGGGATGTGGTATCGAAT |
| Gorai.012G003400-R | ATTCAGACAAAACACACGACTG |
| Gorai.012G014000-F | TAAAGCTGCTTCTTCTTCCTCA |
| Gorai.012G014000-R | CAGCTACTCTCGTTTTGCATTT |
| Gorai.012G119600-F | CGGTAAACTGGAAGAACGATTC |
| Gorai.012G119600-R | GGTTTTCTCTAAACTCCATGCC |
