# Supplementary material for: Embryogenic Calli Induction and Salt Stress Response Revealed by RNA-Seq in Diploid Wild Species Gossypium sturtianum and Gossypium raimondii
Source: Front Plant Sci. 2021 Aug 25;12:715041. doi: 10.3389/fpls.2021.715041 (PMC8424188; doi:10.3389/fpls.2021.715041)
Supplement: Supplementary Figure 1 — Correlation and different expression genes analysis of RNA-seq. (A) Statistical analysis of correlation coefficient between each sample; (B) principal component analysis of each sample; (C) statistics of the number of DEGs in G. raimondii and G. sturtianum; (D,E) Venn diagrams of DEGs in different salt stress stages of G. raimondii (D) and G. sturtianum (E). [file Data_Sheet_1.zip › 20210809-Additional files/Table S9.doc]

**Table S9** List of genes related to MAPK signaling pathway

| Gene ID | Gene Family | log_2_(FoldChange) | | | | | | | |
| --- | --- | --- | --- | --- | --- | --- | --- | --- | --- |
|  |  | R6h vs R0h | R12h vs R0h | R24h vs R0h | R48h vs R0h | S6h vs S0h | S12h vs S0h | S24h vs S0h | S48h vs S0h |
| *Gorai.008G254800* | *NDPK2* | *-1.04* | *-1.05* | *-0.32* | *-0.01* | *-0.27* | **0.02** | **0.41** | **1.04** |
| *Gorai.007G140300* | *CALM* | **1.61** | **2.78** | **2.37** | **1.17** | **4.99** | **2.35** | **2.71** | **0.51** |
| *Gorai.010G165300* | *FLS2* | *-1.55* | *-2.49* | *-2.43* | *-2.47* | *-0.98* | *-1.95* | *-1.89* | *-2.54* |
| *Gorai.012G119600* | *WRKY33* | **1.38** | **1.07** | *-0.10* | *-0.69* | **1.73** | **1.29** | *-0.17* | *-0.03* |
| *Gorai.004G160100* | *WRKY22* | *-0.82* | *-1.07* | *-0.67* | *-0.15* | **1.56** | **0.71** | *-0.07* | **0.74** |
| *Gorai.007G103400* | *WRKY22* | **0.49** | **0.09** | **1.02** | **1.60** | **0.18** | **0.32** | **0.69** | **1.35** |
| *Gorai.001G017400* | *RBOH* | *-1.24* | *-1.18* | *-0.11* | **0.70** | *-1.88* | *-3.39* | *-0.25* | *-5.57* |
| *Gorai.003G085100* | *RBOH* | *-0.08* | **0.32** | **1.37** | **1.35** | *-1.70* | *-1.68* | *-0.80* | *-0.95* |
| *Gorai.007G299500* | *RBOH* | *-1.49* | *-1.84* | *-1.27* | *-0.61* | *-0.19* | *-1.57* | *-1.03* | **0.02** |
| *Gorai.009G202500* | *RBOH* | **1.90** | **2.24** | **2.44** | **2.10** | **3.65** | **3.26** | **3.00** | **1.76** |
| *Gorai.002G266100* | *PYL* | *-0.54* | *-1.41* | *-1.70* | *-1.40* | *-0.62* | *-1.09* | *-1.97* | *-2.28* |
| *Gorai.006G185000* | *PYL* | *-0.97* | *-2.09* | *-3.35* | *-2.90* | *-1.03* | *-1.56* | *-3.79* | *-2.68* |
| *Gorai.007G031800* | *PYL* | *-1.12* | *-1.58* | *-1.67* | *-1.16* | *-0.63* | *-2.09* | *-0.60* | *-2.01* |
| *Gorai.009G323000* | *PYL* | *-1.87* | *-3.80* | *-5.40* | *-4.42* | *-0.26* | *-1.49* | *-2.46* | *-3.17* |
| *Gorai.010G194800* | *PYL* | *-1.57* | *-2.98* | *-3.85* | *-3.42* | *-0.55* | *-1.42* | *-3.41* | *-2.41* |
| *Gorai.011G290300* | *PYL* | *-0.28* | *-1.91* | *-3.41* | *-2.88* | **0.98** | **0.17** | *-1.75* | *-0.74* |
| *Gorai.001G013500* | *PP2C* | **1.17** | **1.47** | **1.24** | **0.85** | **0.83** | **0.28** | **1.03** | *-0.85* |
| *Gorai.003G128900* | *PP2C* | *-2.48* | *-1.82* | *-1.96* | *-2.17* | **2.74** | **0.84** | **1.05** | *-1.58* |
| *Gorai.006G201000* | *PP2C* | **0.20** | **1.00** | **1.49** | **1.29** | **3.01** | **2.28** | **2.91** | **1.57** |
| *Gorai.008G269800* | *PP2C* | **1.71** | **2.97** | **4.17** | **3.53** | **2.05** | *-1.41* | **3.08** | *-0.07* |
| *Gorai.008G282600* | *PP2C* | *-0.24* | **0.90** | **1.39** | **1.11** | **1.02** | **0.68** | **1.48** | **1.25** |
| *Gorai.009G096200* | *PP2C* | **1.73** | **3.29** | **3.25** | **2.22** | **2.08** | **1.45** | **1.98** | **0.17** |
| *Gorai.012G003400* | *PP2C* | **1.78** | **2.86** | **4.31** | **3.45** | **5.01** | **4.59** | **5.45** | **4.46** |
| *Gorai.002G006900* | *SNRK2* | *-1.42* | *-1.22* | *-1.35* | *-1.22* | *-1.63* | *-0.77* | *-0.71* | *-0.88* |
| *Gorai.011G121900* | *SNRK2* | *-1.47* | *-1.65* | *-0.84* | **0.10** | *-1.32* | *-1.51* | *-0.70* | *-0.31* |
| *Gorai.009G304300* | *CTR1* | *-1.22* | *-1.06* | *-0.94* | *-0.27* | *-0.78* | *-1.17* | *-0.65* | *-0.21* |
| *Gorai.004G220800* | *ERF1* | *-0.55* | *-1.31* | *-1.29* | *-2.29* | **2.50** | **1.96** | **1.20** | **1.32** |
| *Gorai.005G049300* | *ERF1* | **0.67** | **0.73** | *-0.56* | *-1.34* | **3.94** | **2.94** | **0.86** | **0.45** |
| *Gorai.009G165500* | *ERF1* | **3.00** | **2.99** | **0.91** | *-1.86* | **11.64** | **7.65** | **7.94** | **0.00** |
| *Gorai.011G173000* | *ERF1* | **0.34** | **0.61** | **1.28** | **0.01** | **2.73** | *-0.72* | **1.23** | *-0.29* |
| *Gorai.004G210800* | *copA* | **0.59** | **0.91** | **1.49** | **1.64** | **0.83** | **1.86** | **1.75** | **0.70** |
| *Gorai.002G203600* | *CHIB* | **0.80** | **1.46** | **1.52** | **1.13** | **1.45** | **1.79** | **1.44** | **1.83** |
| *Gorai.002G019300* | *SUMM2* | *-0.35* | *-1.67* | *-2.08* | *-2.43* | **1.12** | **1.07** | **0.01** | **0.22** |
| *Gorai.001G013600* | *MKK2* | **1.31** | **1.26** | **0.47** | **0.49** | **1.09** | **0.40** | **0.46** | *-0.29* |
| *Gorai.008G291700* | *MKK9* | *-0.89* | *-1.07* | *-1.64* | *-1.77* | *-1.07* | *-1.23* | *-2.44* | *-1.88* |
| *Gorai.N004100* | *OXI1* | *-1.60* | *-1.50* | *-2.02* | *-1.63* | *-1.63* | *-0.09* | *-1.06* | *-0.54* |
| *Gorai.011G116000* | *MKS1* | **1.10** | **0.67** | **0.28** | *-0.49* | **1.66** | **1.48** | **0.55** | **0.45** |
| *Gorai.004G287200* | *TMEM222* | *-0.72* | *-0.83* | *-1.02* | *-0.89* | *-0.32* | *-0.97* | *-1.48* | *-1.01* |
| *Gorai.007G103200* | *ACS1_2_6* | **2.04** | **2.13** | **1.75** | **0.13** | **8.18** | **3.37** | **7.98** | **5.20** |
